# COVID-19 Vaccine Effectiveness and Digital Pandemic Surveillance in Germany (eCOV Study): Web Application–Based Prospective Observational Cohort Study

**DOI:** 10.2196/47070

**Published:** 2024-06-04

**Authors:** Anna-Lena Lang, Nils Hohmuth, Vukašin Višković, Stefan Konigorski, Stefan Scholz, Felix Balzer, Cornelius Remschmidt, Rasmus Leistner

**Affiliations:** 1 d4l Data4Life gGmbH Potsdam Germany; 2 Institute of Medical Informatics Charité University Medicine Berlin Berlin Germany; 3 Digital Health Center Hasso Plattner Institute for Digital Engineering Potsdam Germany; 4 Hasso Plattner Institute for Digital Health Icahn School of Medicine at Mount Sinai New York, NY United States; 5 Department of Statistics Harvard University Cambridge, MA United States; 6 Health Services Research and Health Economics Martin Luther University Halle-Wittenberg Halle Saale Germany; 7 Department of Gastroenterology, Infectiology and Rheumatology Charité University Medicine Berlin Berlin Germany

**Keywords:** COVID-19, SARS-CoV-2, COVID-19 vaccines, BNT162b2, vaccine effectiveness, participatory disease surveillance, web application, digital public health, vaccination, Germany, effectiveness, data collection, disease surveillance, tool

## Abstract

**Background:**

The COVID-19 pandemic posed significant challenges to global health systems. Efficient public health responses required a rapid and secure collection of health data to improve the understanding of SARS-CoV-2 and examine the vaccine effectiveness (VE) and drug safety of the novel COVID-19 vaccines.

**Objective:**

This study (*COVID-19 study on vaccinated and unvaccinated subjects over 16 years*; eCOV study) aims to (1) evaluate the real-world effectiveness of COVID-19 vaccines through a digital participatory surveillance tool and (2) assess the potential of self-reported data for monitoring key parameters of the COVID-19 pandemic in Germany.

**Methods:**

Using a digital study web application, we collected self-reported data between May 1, 2021, and August 1, 2022, to assess VE, test positivity rates, COVID-19 incidence rates, and adverse events after COVID-19 vaccination. Our primary outcome measure was the VE of SARS-CoV-2 vaccines against laboratory-confirmed SARS-CoV-2 infection. The secondary outcome measures included VE against hospitalization and across different SARS-CoV-2 variants, adverse events after vaccination, and symptoms during infection. Logistic regression models adjusted for confounders were used to estimate VE 4 to 48 weeks after the primary vaccination series and after third-dose vaccination. Unvaccinated participants were compared with age- and gender-matched participants who had received 2 doses of BNT162b2 (Pfizer-BioNTech) and those who had received 3 doses of BNT162b2 and were not infected before the last vaccination. To assess the potential of self-reported digital data, the data were compared with official data from public health authorities.

**Results:**

We enrolled 10,077 participants (aged ≥16 y) who contributed 44,786 tests and 5530 symptoms. In this young, primarily female, and digital-literate cohort, VE against infections of any severity waned from 91.2% (95% CI 70.4%-97.4%) at week 4 to 37.2% (95% CI 23.5%-48.5%) at week 48 after the second dose of BNT162b2. A third dose of BNT162b2 increased VE to 67.6% (95% CI 50.3%-78.8%) after 4 weeks. The low number of reported hospitalizations limited our ability to calculate VE against hospitalization. Adverse events after vaccination were consistent with previously published research. Seven-day incidences and test positivity rates reflected the course of the pandemic in Germany when compared with official numbers from the national infectious disease surveillance system.

**Conclusions:**

Our data indicate that COVID-19 vaccinations are safe and effective, and third-dose vaccinations partially restore protection against SARS-CoV-2 infection. The study showcased the successful use of a digital study web application for COVID-19 surveillance and continuous monitoring of VE in Germany, highlighting its potential to accelerate public health decision-making. Addressing biases in digital data collection is vital to ensure the accuracy and reliability of digital solutions as public health tools.

## Introduction

### Background

At the onset of the COVID-19 pandemic in early 2020, the world was far from prepared for pandemic surveillance. Manual case reporting from testing centers and medical facilities to public health authorities was common and remains so even today. However, timely access to accurate disease surveillance data has proven essential for informed public health decision-making and the adaptable response of health care systems [1]. Over the course of the pandemic, numerous innovative digital health solutions [1-8] were developed, focusing on real-time monitoring and predicting the dynamics of the COVID-19 pandemic. The broad adoption of smartphones enabled scalable population-wide data collection [9, 10]. However, recruiting a substantial number of participants for meaningful analyses without the risk of participant bias as well as data privacy concerns remained an ongoing challenge in these digital public health efforts [9].

The most prominent of such solutions developed in Europe were the ZOE COVID Study app in the United Kingdom [2,11,12] and the COVID Symptom Study app in Sweden [1]. Both apps were developed to gather self-reported data for COVID-19 syndromic surveillance on a national scale. The app used in Sweden primarily focused on syndromic surveillance, while the ZOE COVID Study app’s data were also used to test vaccine effectiveness (VE) and drug safety under real-world conditions, as well as to continuously monitor VE as various SARS-CoV-2 variants emerged. The ZOE COVID Study showcased the benefits of using self-reported data to identify COVID-19 hot spots in a timely and efficient manner. It also illustrated how digital apps can continuously monitor VE, providing data for informed decision-making in public health policies. In comparison to phase 3 vaccine efficacy trials, the data from the ZOE COVID Study app showed similar VE and lower frequencies of expected adverse events (AEs) after vaccination and highlighted the importance of second- and third-dose vaccinations for increased protection [2,11,13]. The study conducted in Sweden further confirmed the value of app-based COVID-19 syndromic surveillance. It highlighted the potential of digital health solutions to monitor pandemics at a population level with limited resources, offering a viable approach for addressing public health challenges. Amid varying national strategies, vaccination rates, and COVID-19 prevalence, it remains crucial to evaluate pandemic parameters nationally [14-16].

### Objectives

The objective of this study was to assess the real-world effectiveness of COVID-19 vaccines among the German population, using self-reported data from a web application. The secondary parameters of interest included VE against hospitalization and across different SARS-CoV-2 variants, the frequency of SARS-CoV-2 infections, SARS-CoV-2 testing data over time, and symptom progression. We assessed the validity of the self-reported data gathered in this digital study by comparing these data to official data from public health authorities and nondigital studies. This study highlights the role of digital participatory pandemic surveillance to inform and accelerate efficient public health responses.

## Methods

We followed the STROBE (Strengthening the Reporting of Observational Studies in Epidemiology) guidelines [17] for cohort studies to ensure comprehensive and transparent reporting of our research (Multimedia Appendix 1).

### Study Design

This digital prospective observational cohort study was conducted from May 1, 2021, to August 1, 2022. The study was only promoted in Germany, but the web-based setting of the study facilitated international enrollment. Participants were able to self-enroll in the study. Enrollment was first restricted to individuals aged >18 years. As vaccines were subsequently approved for younger age groups, the study was opened to people aged ≥16 years in December 2021. No further exclusion criteria were applied for self-enrollment. Recruitment was carried out on the web through paid and unpaid marketing on social media platforms and websites as well as offline through advertisements in trains and public spaces, at a mass gathering, and in general practitioner offices across Germany. The study’s offline recruitment was carried out to reduce selection bias and reach a broader range of participants, including individuals who may not have been active on social media platforms. The wording and pictures in the web-based and offline campaigns were targeted to ensure outreach to diverse target groups, including people from different age groups, genders, and professional backgrounds (Multimedia Appendix 2). Demographic information of the study cohort was continuously evaluated for representativeness of the cohort and influenced the ongoing recruitment efforts. Participants could choose to complete the study in German or English.

Participants were asked to complete different questionnaires provided in the study app, which could be accessed on both mobile and desktop devices (Figure S1 in Multimedia Appendix 3). Email notifications reminded participants to respond in a timely manner to any pending questionnaires to minimize recall bias. Participant feedback from an in-app feedback function and qualitative interviews showed that weekly email notifications were perceived as too frequent. Therefore, we switched to monthly email notifications starting December 2021. The email notifications were carefully crafted to ensure neutrality and motivate individuals (regardless of their COVID-19 symptoms or test results) to log in to their accounts and complete the pending questionnaires. This was done to reduce response bias to email notifications.

The digital questionnaires collected demographic information and COVID-19–related data on vaccination status, symptoms, testing, and contact behavior (Multimedia Appendix 4). Study enrollment was defined as creating a user account, agreeing to the terms of use of the study web application, and providing informed digital consent for study participation. Upon enrollment, individuals reported their demographic data and vaccination status information as well as prior SARS-CoV-2 infections. Vaccines available in Germany during the study period were AZD1222 (Oxford-AstraZeneca), BNT162b2 (Pfizer-BioNTech), JNJ-78436735 (Janssen), mRNA-1273 (Moderna), and NVX-CoV2373 (Novavax). COVID-19 tests and symptoms could be reported daily through on-demand questionnaires in the web application. Participants could also report data retrospectively, that is, report a symptom they had experienced in the last month. On a monthly basis, they were asked to provide information on COVID-19 exposure and contact behavior. All answers could be edited within 48 hours. Individuals who experienced AEs after vaccination were able to report them through the certified web-based pharmacovigilance platform Medikura [18]. Medikura forwarded all reports on AEs to the EudraVigilance database hosted by the European Medicines Agency [19]. Under a contractual agreement with Medikura, we were provided with data from our study participants for the purposes of this study. By design, the collected data were pseudonymized and not linked to study participants in our data set.

### Vaccination Status

On the basis of the self-reported data, study participants were grouped by vaccination status for downstream analysis. We differentiated between the total number of vaccine doses received and the combination of types of vaccines. For our main VE analysis, we focused on 2 specific groups: unvaccinated participants (1768/9287, 19.04%) and individuals who completed the primary vaccination series with 2 doses of BNT162b2 and had no prior infections before vaccination (3463/9287, 37.29%).

### Outcomes

Our primary outcome measure for this study was the effectiveness of SARS-CoV-2 vaccines against laboratory-confirmed (polymerase chain reaction [PCR] test) SARS-CoV-2 infection, independent of any current symptomatology. The secondary outcome measures included the effectiveness of SARS-CoV-2 vaccines against symptomatic infection, against severe infection defined by hospitalization, and VE across different SARS-CoV-2 variants. We further evaluated COVID-19 symptoms over time and by variant and investigated self-reported AEs possibly associated with the SARS-CoV-2 vaccinations. To assess the validity of the self-reported data, we compared the data with official surveillance data from public health authorities, focusing on COVID-19 vaccine coverage, test positivity rates, and 7-day incidence.

### Case Definitions

Study participants’ exposure to COVID-19 was based on results from self-reported PCR tests. An individual was classified as a *confirmed case* if a positive PCR test was reported, following the definition of SARS-CoV-2 infection used by the German public health authorities [20]. Reinfection was only considered if it occurred >60 days after the last infection. An infection was considered symptomatic if any symptoms occurred within 5 days before the first positive test to 10 days after it. By design, participants were not required to get tested and submit test results on a regular basis but rather on demand. Not reporting a SARS-CoV-2 test was thus weighted as equal to not being infected. To reduce reporting bias, monthly email notifications invited individuals to answer the pending questionnaires independent of symptomatology.

### Data Source

The *COVID-19 study on vaccinated and unvaccinated subjects over 16 years* (eCOV study) was registered with the German Clinical Trials Register (DRKS00025169). The acronym “*eCOV*” was selected to reflect the electronic (“*e*”) or digital nature of this study on COVID-19 (“*COV*”). The study and the web application were developed by the nonprofit organization Data4Life, based in Potsdam, Germany. The study app was a web application that was accessible on both desktop and mobile devices (Figure S1 in Multimedia Appendix 3). Self-reported data were collected via the study app and processed by Data4Life’s General Data Protection Regulation–compliant research infrastructure, certified by the German Federal Office for Information Security. Data from two other data sources were collected to monitor and analyze web application log-ins and email notifications: (1) logs generated from the study app; and (2) if the participant had provided consent to place optional cookies, data generated from the General Data Protection Regulation–compliant web analytics tool Matomo [21]. The logs generated from the study app enabled the analysis on daily log-ins and responses to email notifications. Data from Matomo enabled the analysis on device types used for log-ins and participation.

### Quality Control

Quality control of the self-reported data included removing individuals from the cohort who reported unlikely dates of vaccination. We thus removed participants if no real date was reported, the date of vaccination was before the first possible vaccination date in Germany in December 2021, participants reported an impossible order of vaccination dates, and if vaccine dates lay within <2 weeks of each other. Participants were further removed from the cohort if the reported vaccine type was *unknown*, *other*, or *Sputnik V*. If the same vaccination questionnaire was submitted multiple times with differing vaccine dates, the first reported date was considered. Apart from this, whenever questionnaires were answered multiple times, or answers were edited by study participants, the last reported answer was considered for analysis.

### Statistical Analysis

All analysis was performed on the Data4Life analytics platform, hosting a JupyterHub notebook using *pandas* (version 1.4.2) [22], *matplotlib* (version 3.5.2) [23], *seaborn* (version 0.11.2) [24], and *numpy* (version 1.22.4) [25]. Descriptive statistics were used to report details about the study cohort and the respondents’ reporting behavior. Continuous values were reported as mean and SD, and categorical values were reported as numbers with percentages. The chi-square test of independence was used for a difference in the numbers of symptoms between vaccinated and unvaccinated individuals. The Shapiro-Wilk test was used to test for normal distribution of the data. We used the Mann-Whitney *U* test for not normally distributed data to compare the 7-day incidences of COVID-19 among groups and the number of reported tests.

VE analysis was carried out as a measure of how well vaccination protects against the defined outcome. For VE analysis, we ran an intention-to-treat analysis protocol, including individuals in the analysis independent of their study dropout date. As the questionnaires administered upon enrollment included retrospective questions about prior vaccinations and infections, data from early dropouts were still valuable for VE analysis. VE was calculated for individuals who received 2 doses of any vaccine brand as well as for individuals who received 2 or 3 doses of BNT162b2. We calculated VE for 4 to 48 weeks after the vaccine dose of interest. If participants received >2 vaccine doses, data were only considered until the time point of the next vaccination. In preparation for logistic regression analyses, each vaccinated person was matched with an unvaccinated person of the same age group (−5 y to +5 y) and gender who had submitted data within the same time frame. Subsequently, a binary logistic regression model was used to obtain the odds ratios of SARS-CoV-2 infections in the respective time frames of interest, given the vaccination status. As the primary outcome of interest, we analyzed SARS-CoV-2 infection, regardless of symptomatology. We adjusted for comorbidity (binary variable, with or without comorbidities), health care worker status (binary variable), age, and age-adjusted 7-day incidence in the German population during the time frame of interest. These covariates were added to the logistic regression model following theoretical criteria using backward elimination. For the secondary outcomes—VE against symptomatic infections, VE by variant, and VE against hospitalization—the same covariates were added to the logistic regression model. Next, the logit model was fitted using the *Newton-Raphson* optimization method implemented in the *statsmodels* Python library [26], accounting for matched pairs. Adjusted VE was then calculated as 1–odds ratio. One logistic regression model was run for each time frame of interest. To consider the effect of different SARS-CoV-2 variants on VE, we defined periods of variant dominance. In Germany, the Delta variant was dominant starting calendar week 26, 2021, and the Omicron variant was dominant starting calendar week 2, 2022 [27]. Only individuals vaccinated during the dominance periods of the respective variants were considered and matched with unvaccinated individuals who had contributed data in the same time frame. To assess the comparability of our results and the influence of covariates added to the logistic regression models, we used the Farrington screening method to calculate unadjusted VE and 95% CIs using the same matching principle [28,29].

### Ethical Considerations

The study was approved by the ethics committee of the Berlin Chamber of Physicians (Eth-11/22). Registration was open for people aged ≥16 years. All participants provided digital informed consent for study participation. Participation was voluntary, and no incentives were offered. Email address and password as well as 2-factor authentication were required for log-in. Individuals could access all study content through a web application on both desktop and mobile devices. All study data were end-to-end encrypted and pseudonymized. Only authorized researchers were provided access to the data on the Data4Life analytics platform. Data were stored exclusively at Data4Life data centers in Germany. Data4Life is certified by the German Federal Office for Information Security on the basis of IT-Grundschutz (ISO 27001).

## Results

### Study Setting and Participants

A total of 10,077 participants self-enrolled in this digital cohort study. After thorough quality control (Figure S2 in Multimedia Appendix 3), data from 9287 (92.16%) of the 10,077 participants were available for final analyses presented in this paper (Table 1). Most of the study participants were women (6567/9287, 70.71%), while 0.32% (30/9287) identified as nonbinary. Study participation was not limited to German residents, but because the study was mainly advertised in Germany, only a few people participated from abroad (755/9287, 8.13%). Concerning possible risk factors for infections, slightly more than a quarter (2463/9287, 26.52%) of the participants reported living with allergies, while 5.69% (529/9287) had an immune deficiency, and 19.96% (1854/9287) reported living with other chronic health conditions. Active smokers made up 16.7% (1551/9287) of the study population, and the average BMI was 25 (SD 5.6) kg/m^2^. As the study was also advertised at a mass gathering for medical professionals, a large proportion (3610/9287, 38.87%) of the study population hailed from the health care sector, mainly hospital employees (1046/3610, 29%) or medical students (1258/3610, 34.85%). At study end (August 1, 2022), of the 9287 participants, 1768 (19.04%) were unvaccinated, 1801 (19.39%) had reported 1 vaccine dose, 3119 (33.58%) had reported 2 doses, 2433 (26.19%) had reported 3 doses (commonly referred to as the first *booster* vaccination), 164 (1.77%) had reported 4 doses, and 2 (0.02%) had reported 5 doses.

**Table 1:** Descriptive characteristics of the study cohort (n=9287).

Characteristics			Values
Age (y), mean (SD)			37.3 (14.9)
**Gender, n (%)**			
	Woman	6567 (70.71)	
	Man	2658 (28.62)	
	Nonbinary	30 (0.32)	
**Residency, n (%)**			
	Germany	8532 (91.87)	
	Abroad	755 (8.13)	
**Health status, n (%)**			
	Immune deficiency	529 (5.69)	
	Allergies	2463 (26.52)	
	Other chronic diseases	1854 (19.96)	
Active smoker, n (%)			1551 (16.7)
BMI (kg/m^2^), mean (SD)			25 (5.6)
**Profession, n (%)**			
	Not health care sector	4647 (50.03)	
	Health care sector	3610 (38.87)	
	Physician’s office	248 (2.67)	
	Nursing facility or retirement home	294 (3.16)	
	Other medical field	764 (8.22)	
	Medical student	1258 (14.62)	
	Hospital	1046 (11.26)	
**Vaccine status at study end, n (%)**			
	Unvaccinated	1768 (19.04)	
	1 dose	1801 (19.39)	
	2 doses	3119 (33.58	
	3 doses	2433 (26.19)	
	4 doses	164 (1.77)	
	5 doses	2 (0.02)	

### Reporting Behavior and Retention

Over the course of the study, participants reported a total of 44,786 tests and 5530 symptoms. With 4263 (45.9%) of the 9287 participants leaving the study within 1 week after enrollment, the average length of participation was 10 weeks (mean 70.2, SD 105.6 days). Individuals who stayed in the study for >1 week (5024/9287, 54.1%) stayed for an average of 18.4 weeks (mean 129.3, SD 113.0 days). Individuals leaving the study after 1 week were still considered in the analysis because the questionnaires administered upon enrollment included retrospective questions about prior vaccinations and infections. An average of 145.7 (SD 324.5) daily log-ins were observed during the study period, of which an average of 111.6 (SD 312.2) were observed after an email notification containing reminders about study participation (Figure S3 in Multimedia Appendix 3). Daily log-ins were always the highest in the first 48 hours after email notifications (Figure S3 in Multimedia Appendix 3). Most log ins were conducted from mobile devices (18,547/23,865, 77.71%).

### VE Against SARS-CoV-2 Infection of Any Severity

At study end, approximately three-fourths of the nationally distributed vaccine doses in Germany were BNT162b2 [30], explaining the low sample size for other vaccine brands in our cohort (Multimedia Appendix 5). Therefore, our primary VE analysis focused on 2 specific groups: unvaccinated participants (1768/9287, 19.04%) and individuals who completed the primary vaccination series with 2 doses of BNT162b2 and had no prior infections before vaccination (3463/9287, 37.29%). The participants’ ages—unvaccinated: mean 38.9 (SD 13.3) years and vaccinated: mean 37.2 (SD 15.0) years—did not differ substantially by vaccination status (Table 2). The majority of the participants in the 2 groups—unvaccinated: 69.62% (1231/1768) and vaccinated: 73.08% (2531/3463)—identified as women. Study participation was not limited to German residents, but because the study was mainly advertised in Germany, only a few participants—unvaccinated: 14.03% (248/1768) and vaccinated: 6.12% (212/3463)—resided outside Germany. Both groups showed similar risk profiles in terms of BMI and comorbidities. More health care workers were among those vaccinated (1428/3463, 41.23%), whereas more active smokers (417/1768, 23.58%) were among those unvaccinated (Table 2). Relatively fewer infections were reported after receiving 2 doses of BNT162b2 (992/3463, 28.64%) compared with unvaccinated individuals during the study period (637/1768, 36.02%; chi-square test, *P*<.001). However, vaccinated individuals were more likely to report symptoms associated with infections (chi-square test, *P*=.02) and reported more tests (mean 5.33, SD 10.6) than unvaccinated individuals (mean 5.02, SD 13.0; Mann-Whitney *U* test, *P*<.001; Table 2). Response rates to monthly questionnaires showed a substantial difference between vaccinated and unvaccinated individuals: only 38.85% (687/1768) of the unvaccinated individuals completed at least 1 monthly questionnaire asking about exposure to COVID-19 and infections in past weeks, whereas 65.2% (2258/3463) of the vaccinated individuals did so.

**Figure 1:**
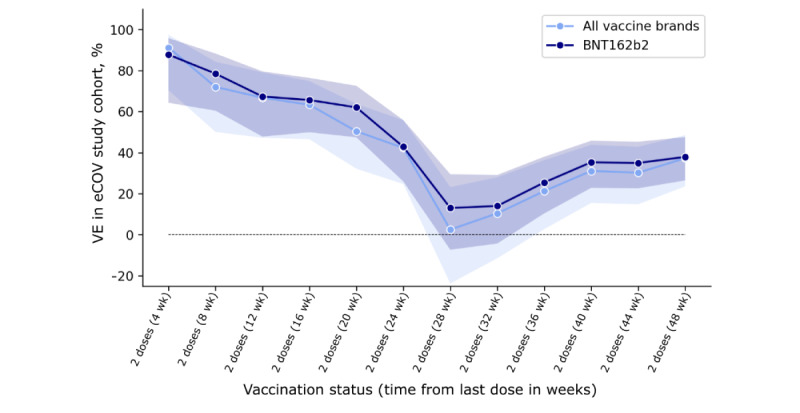
Vaccine effectiveness (VE) against infection of any severity in the COVID-19 study on vaccinated and unvaccinated subjects over 16 years (eCOV study) cohort in the weeks after the second dose of BNT162b2. VE (%) was calculated for weeks 4 to 48 after completing the primary vaccination series with 2 doses of BNT162b2 (Pfizer-BioNTech). Shaded areas indicate 95% CIs.

We calculated VE against infection across all SARS-CoV-2 variants and for the Delta variant separately using logistic regression models (refer to the *Statistical Analysis* subsection under Methods). Across all vaccine brand combinations, VE against infection of any severity 4 weeks after the second dose was 87.8% (95% CI 64.3%-95.8%), which waned to 42.8% (95% CI 26%-55.8%) after 24 weeks and 37.9% (95% CI 26.5%-47.6%) after 48 weeks (Figure 1; Table 3). Similar numbers were seen for VE after 2 doses of BNT162b2: after 4 weeks, VE was 91.2% (95% CI 70.4%-97.4%), which decreased to 42.3% (95% CI 24.7%-55.8%) after 24 weeks. VE then declined in weeks 28 to 32 after the second dose (Figure 1; Tables 3 and 4) before increasing to 37.2% (95% CI 23.5%-48.5%) after 48 weeks. Similar results were seen using the Farrington screening method [28] to calculate unadjusted VE (Table 4. VE was increased to 67.6% (95% CI 50.3%-78.8%) at 4 weeks after the third dose of BNT162b2 (Figure S4 in Multimedia Appendix 3).

**Table 2:** Descriptive characteristics of the study population split by vaccination status. Infections among unvaccinated individuals were compared with those among individuals who received at least 2 doses of BNT162b2 (Pfizer-BioNTech) and did not have any prior infections before vaccination.

Characteristics				Unvaccinated individuals (n=1768)		Vaccinated individuals (2 doses of BNT162b2; n=3463)
Age (y), mean (SD)				38.9 (13.3)		37.3 (15.0)
**Gender, n (%)**						
	Woman		1231 (69.63)		2531 (73.1)	
	Man		514 (29.07)		921 (26.6)	
	Nonbinary		6 (0.34)		10 (0.28)	
**Residency, n (%)**						
	Germany		1520 (85.97)		3252 (93.9)	
	**Abroad**		248 (14.03)		212 (6.12)	
		Austria	162 (9.16)		148 (4.27)	
		Switzerland	36 (2.03)		23 (0.66)	
		Other	50 (2.83		41 (1.18)	
**Any chronic disease, n (%)**						
	Allergies		418 (23.64)		959 (27.69)	
	Others		348 (19.68)		721 (20.82	
Immune deficiency, n (%)				118 (6.67)		206 (5.95)
Active smoker, n (%)				417 (23.59)		519 (14.99)
Health care worker, n (%)				388 (21.95)		1428 (41.24)
BMI (kg/m^2^), mean (SD)				25.4 (6.3)		25 (5.5)
Infections^a^, n (%)				637 (36.03)		992 (28.65)^b^
Symptomatic infections, n (%)				318 (17.99)^c^		734 (21.2)
Test reports per person, mean (SD)				5.0 (13.0)		5.3 (10.6)^d^

^a^Infections after vaccination for vaccinated individuals and infections over the study period for unvaccinated individuals.

^b^Chi-square test, *P*<.001.

^c^Chi-square test, *P*=.02.

^d^Mann-Whitney *U* test, *P*<.001.

**Table 3:** Vaccine effectiveness against infection of any severity after the second vaccine dose across all SARS-CoV-2 variants. The numbers only reflect vaccine effectiveness against infection, independent of symptoms.

Weeks after last dose	Second dose, any vaccine brand (%, 95% CI)	Second dose, BNT162b2 (Pfizer-BioNTech; %, 95% CI)
4	87.8 (64.3 to 95.8)	91.2 (70.4 to 97.4)
8	78.5 (60.5 to 88.3)	72 (50.1 to 84.3)
12	67.4 (47.8 to 79.6)	66.8 (47.2 to 79.1)
16	65.6 (49.9 to 76.4)	63.3 (46.5 to 74.8)
20	62.1 (47.5 to 72.6)	50.4 (23.1 to 63.7)
24	42.8 (26 to 55.8)	42.3 (24.7 to 55.8)
28	13 (7.3 to 29.5)	2.5 (−23.8 to 23.1)
32	14 (4.3 to 29.1)	10.4 (−11.5 to 28)
36	25.5 (10.4 to 38)	21.3 (2.7 to 36.3)
40	35.3 (22.8 to 45.8)	31.1 (15.5 to 43.8)
44	34.9 (22.6 to 45.3)	30.2 (14.8 to 42.8)
48	37.9 (26.5 to 47.6)	37.2 (23.5 to 48.5)

**Table 4:** Vaccine effectiveness (VE) against infection of any severity after the second dose of BNT162b2 (Pfizer-BioNTech) across all SARS-CoV-2 variants. The numbers only reflect VE against infection, independent of symptoms. Adjusted VE was calculated using a logistic regression model with adjustment for age, comorbidity, health worker status, and age-adjusted average incidence in the German population during the time frame of interest. Unadjusted VE was calculated using the Farrington screening method.

Weeks after second vaccine dose	Sample size, n	Cases (positive PCR^a^ test), n (%)		Adjusted VE (logistic regression; %, 95% CI)	Unadjusted VE (Farrington method; %, 95% CI)
		Vaccinated individuals	Unvaccinated individuals		
4	1679	3 (0.2)	33 (2)	91.2 (70.4 to 97.4)	90.9 (70.4 to 97.2)
8	1680	17 (1)	55 (3.3)	72 (50.1 to 84.3)	69.1 (46.8 to 82.1)
12	1679	32 (1.9)	79 (4.7)	66.8 (47.2 to 79.1)	59.5 (38.9 to 73.1)
16	1678	49 (2.9)	113 (6.7)	63.3 (46.5 to 74.8)	56.6 (39.4 to 69)
20	1680	78 (4.6)	142 (8.5)	50.4 (23.1 to 63.7)	45.1 (27.6 to 58.3)
24	1658	118 (7.1)	184 (11.1)	42.3 (24.7 to 55.8)	35.9 (19.2 to 49.1)
28	1559	174 (11.2)	187 (12)	2.5 (−23.8 to 23.1)	7 (−14.4 to 24.3)
32	1446	213 (14.7)	241 (16.7)	10.4 (−11.5 to 28)	11.6 (−6.3 to 26.5)
36	1391	219 (15.7)	272 (19.6)	21.3 (2.7 to 36.3)	19.5 (3.8 to 32.6)
40	1355	231 (17)	309 (22.8)	31.1 (15.5 to 43.8)	25.2 (11.3 to 37)
44	1314	238 (18.1)	320 (24.4)	30.2 (14.8 to 42.8)	25.6 (12 to 37.1)
48	1319	233 (17.7)	343 (26)	37.2 (23.5 to 48.5)	32.1 (19.8 to 42.5)

^a^PCR: polymerase chain reaction.

### VE by SARS-CoV-2 Variant

To further inspect the influence of the SARS-CoV-2 Delta variant on VE, we filtered the data set for individuals who received their second vaccine dose during the dominance period of the Delta variant in Germany (1363/3463, 39.36%). In this case, 4 weeks after the second dose of BNT162b2 was administered, the VE against infection was 76.8% (95% CI 12.5%-93.8%), which decreased to 40.6% (95% CI 8%-61.76%) at 24 weeks (Figure S5 in Multimedia Appendix 3). The sample size of vaccinated individuals reporting data after week 24 was too low (206/3463, 5.95%). Furthermore, we were underpowered for analysis of VE for the SARS-CoV-2 Omicron variant because only 30 (0.87%) of the 3463 participants vaccinated with BNT162b2 completed their primary immunization series with BNT162b2 during the dominance period of the Omicron variant.

### VE Against Symptomatic SARS-CoV-2 Infection

As the number of symptomatic infections in our study was low (1563/2834, 55.15%), the CIs of the estimated VE against symptomatic infection were correspondingly large (Multimedia Appendix 6). VE against symptomatic infection in the eCOV study cohort seemed to be lower after vaccination than VE against infection of any severity (Figure S6 in Multimedia Appendix 3).

### Time Between SARS-CoV-2 Infections Was Prolonged in Vaccinated Individuals

Among the 2834 PCR test–confirmed COVID-19 cases, 90 (3.2%) individuals reported ≥2 infections. Among these 90 individuals, 32 (36%) were unvaccinated, and 23 (26%) had been vaccinated twice with any vaccine type at the time of both infections. The median time between the first and second infections for the unvaccinated individuals was 159.0 (IQR 106.25-362.0) days, and for those who had been vaccinated twice, it was 385.5 (IQR 342.0-438.5) days (Mann-Whitney *U* test, *P*<.001).

### Pain at the Injection Site Was the Most Reported AE After Vaccination

A total of 16,004 vaccine doses were reported during the study. The question of whether expected or unexpected AEs occurred after vaccination was answered 15,363 times by 95.04% (7147/7519) of those who had been vaccinated at least once. The reported incidence of AEs decreased slightly with the increasing number of vaccinations, from 81.45% (5821/7147) after the first dose to 72.49% (4007/5528) after the second dose and 71.35% (1659/2325) after the third dose of any vaccine brand. In addition to categorical *yes* or *no* responses to suspected AEs, 2364 reports were submitted that included a total of 7373 expected and unexpected AEs after vaccination. As most AEs are expected within 6 weeks after vaccination, we reported only AEs occurring in this time frame (3988/7373, 54.08%). Of these AEs, 48.84% (1948/3988) occurred after the first dose, 31.24% (1246/3988) after the second dose, and 17.3% (690/3988) after the third dose. Across all vaccine brands and regardless of the number of doses received, local pain or tenderness at the injection site was the most common expected AE after vaccination (697/3988, 17.47% of all AEs), followed by headache (665/3988, 16.67%), fatigue or exhaustion (657/3988, 16.47%), body ache (459/3988, 11.5%), and fever (376/3988, 9.42%). Among the local AEs (822/3988, 20.61%), pain or tenderness at the injection site was the most common, (697/822, 84.8%), followed by swelling or pain of the lymph nodes (69/822, 8.4%; Figure S7A in Multimedia Appendix 3). Headache was the leading systemic AE (665/3166, 21%), followed by fatigue and exhaustion, body ache, and fever (Figure S7B in Multimedia Appendix 3). The distribution of local and systemic AEs did not differ notably by number of doses and vaccine brands (Figures S8 and S9 in Multimedia Appendix 3). Apart from the expected AEs, the following serious unexpected AEs were reported within 6 weeks of vaccination: 1 cerebral sinus thrombosis, 2 reports of anaphylaxis (not further specified), and 3 reports of hemiplegia and paralysis of the face (facial nerve palsy). We do not know whether the reported AEs were attributable to the vaccination because the reporting was anonymous, and we were not involved in follow-up investigations. All AEs collected by our study partner Medikura were reported to the EudraVigilance database hosted by the European Medicines Agency. We cannot comment on AEs associated with different vaccine combinations because this information was not available to us.

### Loss of Taste or Smell Was the Most Frequent Symptom During SARS-CoV-2 Infection

Of the 2834 PCR test–confirmed COVID-19 cases reported to our app, 1563 (55.15%) infections were reportedly symptomatic. A total of 3374 symptoms were reported within 5 days before the first positive PCR test to 10 days after it. While 74.1% (734/992) of the infections in individuals who had received 2 doses of BNT162b2 were reportedly symptomatic, only 49.9% (318/637) of the infections in unvaccinated individuals were reportedly symptomatic. The most frequent symptom accompanying infection, regardless of vaccination status and SARS-CoV-2 variant, was the loss of taste or smell, which was reported in 26.4% (413/1563) of all PCR test–confirmed symptomatic infections and, during the dominance period of the Delta variant, in 54.5% (165/303) of all PCR test–confirmed symptomatic infections (Multimedia Appendix 7), followed by fever (343/1563, 21.94%), cough (299/1563, 19.12%), and diarrhea (271/1563, 17.34%). Across all SARS-CoV-2 variants, infected individuals primarily vaccinated with 2 doses of BNT162b2 reported fewer occurrences of loss of taste or smell (177/744, 23.8%) than unvaccinated individuals (114/364, 31.3%; chi-square test, *P*=.009). This difference was most notable in infections during the dominance period of the Delta variant and disappeared during the dominance period of the Omicron variant, when only 18.29% (217/1186) of all symptomatic infections were associated with loss of taste or smell as a symptom, with similar distribution in vaccinated and unvaccinated individuals (Multimedia Appendix 7). Notably, cough during infection was reported more often by individuals vaccinated with 2 doses of BNT162b2 (163/744, 21.9%) compared to unvaccinated individuals (28/364, 7.7%, chi-square test *P*<.001). Of the 1563 individuals with symptomatic infections, 397 (25.4%) received medical treatment, and 20 (1.28%) were hospitalized. The hospitalization rate for symptomatic infections was 2.47% (9/364) among unvaccinated individuals and 0.8% (6/744) among individuals who had received 2 doses of BNT162b2 before infection (chi-square test, *P*=.048; Multimedia Appendix 8). Notably, the average age as a highly influential factor for hospitalization did not differ significantly between the 2 groups.

### Vaccine Coverage for Primary Vaccination Series Shows Representative Timeline

**Figure 2:**
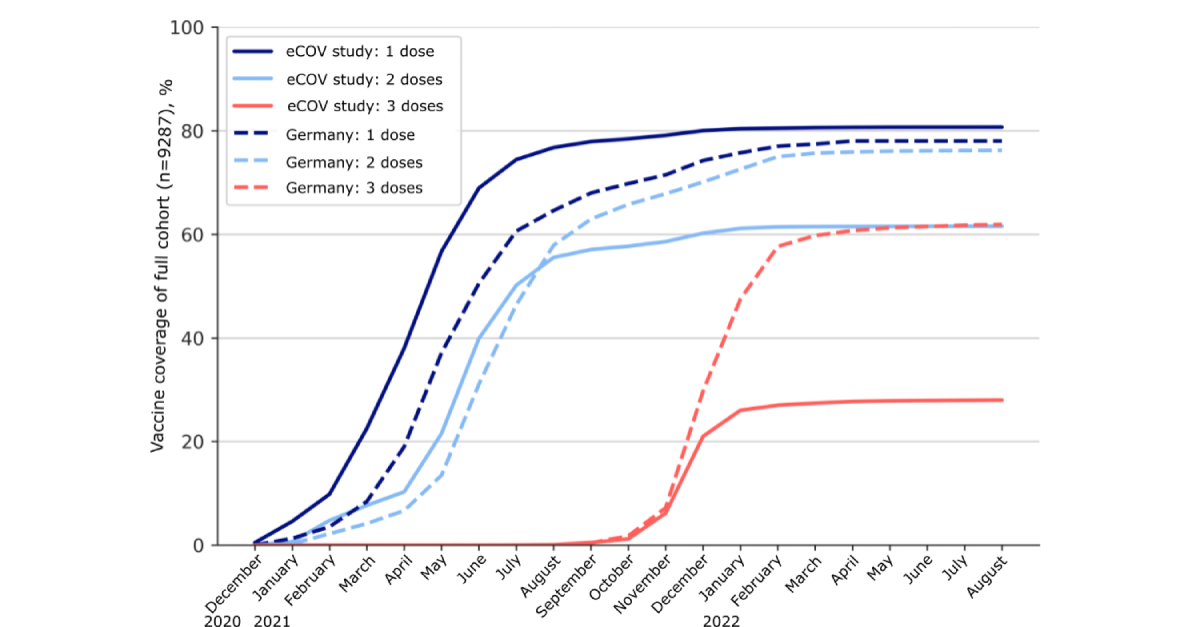
Vaccine coverage over time. Line plot visualizing the vaccine coverage over time within the COVID-19 study on vaccinated and unvaccinated subjects over 16 years (eCOV study) cohort from the first possible COVID-19 vaccination date in Germany (December 26, 2020) to study end (August 1, 2022). Data are split and color coded by the number of vaccines received. The vaccine coverage data for first, second, and third vaccine doses among the German population are provided as a reference as a dashed line.

The coverage for the first vaccine dose (7496/9287, 80.71%) in the eCOV study cohort showed a representative timeline, with slightly higher coverage at study end compared to the German population (78%) [30]. The percentage of eCOV study participants who received 2 doses of any vaccine increased to 61.58% (5719/9287) until study end in August 2022, which was below the vaccine coverage in the German population (76.2%; Figure 2), although the data from Germany were not matched by age in this comparison [30]. Notably, 71.4% (1286/1801) of the individuals who only reported 1 vaccination until study end dropped out after 1 week, which explains the lower percentage of vaccine coverage for 2 vaccines in our cohort compared to population data from Germany. The highest vaccine coverage for 2 doses in our cohort was seen in the younger age group (20-29 y; 2483/3764, 65.96%) and in individuals aged >60 years (630/930, 67%; Figure S10 in Multimedia Appendix 3). A high percentage (2396/3764, 63.65%) of the younger eCOV study participants were health care workers, which explains early access to vaccination. The early access to vaccination and high vaccine coverage among individuals aged >60 years possibly reflect the prioritized distribution of vaccines among older adults in Germany. At study end, only 27.98% (2599/9287) of the eCOV study cohort reported that they had received at least 3 vaccine doses, compared to the booster coverage of 61.9% in the German population during that time, which can be explained with study dropouts over time (refer to the *Reporting Behavior and Retention* subsection in this section).

Regarding the distribution of vaccine brands in our cohort, the majority of individuals who received 2 vaccine doses were vaccinated with BNT162b2 (3561/5718, 62.27%; Multimedia Appendix 5), while 0.59% (34/5718) of the individuals did not report the brand of their second vaccine dose. Among individuals who received a booster dose, 3 doses of BNT162b2 were again most common (1167/2587, 45.11%; Multimedia Appendix 9). The mean time between administered doses was 50 (SD 37.4) days between the first and second doses, 187 (SD 44.8) days between the second and third does, and 150 (SD 55.8) days between the third and fourth does.

### Test Positivity Rate in the eCOV Study Cohort Was Higher Than Germany-Wide Numbers

**Figure 3:**
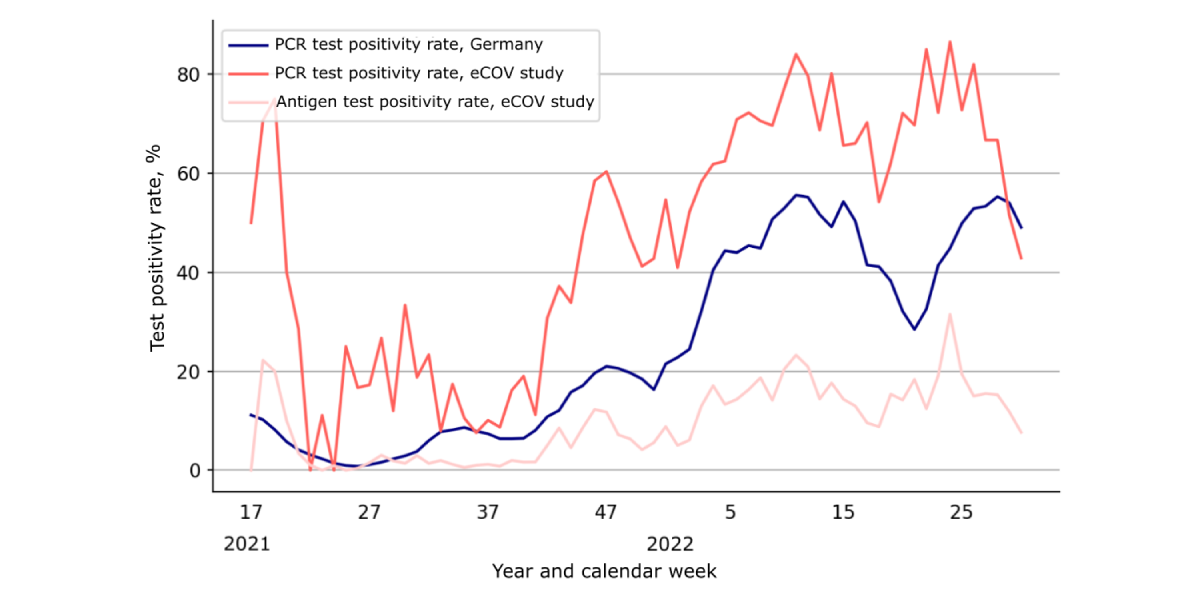
Test positivity rates in the COVID-19 study on vaccinated and unvaccinated subjects over 16 years (eCOV study) cohort and the German population over time. Calendar week 17, 2021, marks the start of the eCOV study in May 2021. PCR: polymerase chain reaction.

The test positivity rate for PCR testing in the eCOV study cohort mirrored the time course of the respective data for the test positivity rate in the German population, with peak rates in 2022 around calendar weeks 7 to 13 (dominant variant: Omicron BA.1) and calendar weeks 22 to 25 (dominant variant: Omicron BA.5). The test positivity rate in the eCOV study cohort was mostly higher compared to not–age-matched data from the German population over time (Figure 2) [31]. The peak around calendar week 18 in 2021 can be explained with the low sample size (58/9287, 0.62%) at the beginning of the study. For antigen tests reported in our cohort, the test positivity rate reflected the course of the pandemic but was lower compared with PCR positivity rates (Figure 3).

### Self-Reported SARS-CoV-2 Infections Reflected the Peak Incidences in the German Population

**Figure 4:**
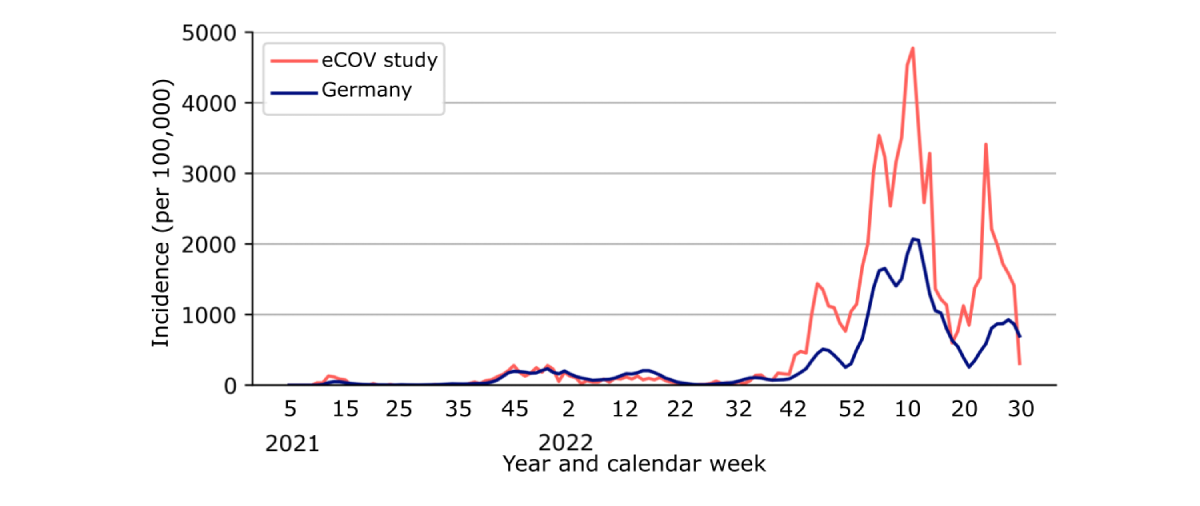
Comparison of 7-day incidence per 100,000 people in the COVID-19 study on vaccinated and unvaccinated subjects over 16 years (eCOV study) cohort and the German population during the study period from May 1, 2021, to August 1, 2022. The x-axis shows the timeline in calendar weeks from calendar week 5, 2021, to calendar week 30, 2022. The data for Germany consisted of the official figures reported by the national infectious disease surveillance system (refer to the Methods section).

Over the course of the study, we registered a total of 2834 PCR test–confirmed SARS-CoV-2 infections from 2743 participants. The demographics of these individuals split by COVID-19 case status, independent of vaccine status, can be found in Multimedia Appendix 10. The trajectory of COVID-19 incidence in the eCOV study cohort aligned with the age-matched Germany-wide incidences registered by public health authorities (Figure 3) [32]. Starting in calendar week 38 in 2021, the 7-day incidence in the eCOV study cohort was consistently above the Germany-wide 7-day incidence for the same age groups (Figure 4). Except for a high incidence peak during the Omicron wave at the beginning of 2022, incidences did not differ substantially between health care workers and participants not working in health care (Mann-Whitney *U* test, *P*=.56; Figure S11 in Multimedia Appendix 3). In addition, the number of reported tests did not differ between health care workers and individuals who did not work in the health care sector (Figure S12 in Multimedia Appendix 3). Overall, peak 7-day incidences were the highest during the dominance period of the Omicron variant, with the highest incidences among individuals aged 20 to 59 years (Figure S13 in Multimedia Appendix 3).

## Discussion

### Principal Findings

To our knowledge, this is the first digital cohort study to collect population-level data related to the COVID-19 pandemic in Germany, focusing on the real-world effectiveness of COVID-19 vaccines. We observed a gradual decline in VE against infection over time, especially in the context of the SARS-CoV-2 Omicron variant. Furthermore, we saw lower hospitalization rates among vaccinated individuals. Booster vaccinations were found to partially restore VE. The reported expected AEs, both local and systemic, aligned with results from previously published findings. Our self-reported digital data closely mirrored official surveillance trends, indicating the utility of digital apps in tracking pandemic waves. Notably, we identified potential biases, such as differences in reporting behavior between vaccinated and unvaccinated individuals, underscoring the importance of optimal study designs and the meticulous interpretation of self-reported data.

With respect to our primary outcome, the study provides valuable insights into the real-world effectiveness of COVID-19 vaccines. Our data suggest that the immunity of this young, mainly healthy, and primarily female cohort waned substantially 24 weeks after primary vaccination series with any vaccine combination and with BNT162b2. Thus, we saw a faster waning compared to previously published results from the ZOE COVID Study app in the United Kingdom [1], where VE was still hovering at 75.7% at 8 months (approximately 32 weeks) after the second dose of BNT162b2 compared to 10.4% (95% CI −11.5% to 28%) in the eCOV study cohort. The waning of VE over time was comparable to results from Qatar [33], where VE dropped to <20% after 24 weeks, and to Sweden [1], where VE waned from 92% at 4 weeks to 23% at 30 weeks. By contrast, in a multinational, placebo-controlled, observer-blinded, pivotal efficacy trial, VE against infection of any severity stayed at >90% at 24 weeks after the second vaccine dose [34]. The waning effect seen in our cohort could additionally have been influenced by the dominance period of the Omicron variant in Germany starting January 2022 because by then most of the vaccinated participants (4052/5718, 79.86%) had already received their second vaccine dose >24 weeks previously. Notably, VE in our cohort was not steadily decreasing but increasing again starting week 36 after the second dose—an observation that could also have been influenced by reporting behavior in our self-selected cohort. Vaccinations could potentially not have been reported among the unvaccinated participants, or booster effects could have occurred through undetected natural infections. We ran several sensitivity analyses adding different covariates to the logistic regression models to check for confounders. Health worker status seemed to negatively impact VE results in weeks 28 to 36 (Figure S14 in Multimedia Appendix 3).

With regard to the secondary outcome measures of this study, exploring VE of third-dose (booster) vaccinations, our data suggest that VE against infection was increased in the first weeks after the third vaccine dose, highlighting the importance of booster vaccinations to restore protection [2,35,36,37]. Interestingly, starting week 20 after the third dose, VE dropped below 0%. As most individuals in the eCOV study cohort received their third vaccination in the time frame from November 2021 to January 2022, this again coincides with the spread of the Omicron variant in Germany. Our data therefore possibly reflect the known reduced protection of vaccination against (symptomatic) infection with the Omicron variant [38], but several other forms of bias may have played a role here. By design, we did not require individuals to get tested and submit test results on a regular basis but rather on demand. Not reporting a SARS-CoV-2 test was thus weighted as equal to not being infected. As it can be assumed that testing behavior is different in vaccinated and unvaccinated individuals [39], infections could have stayed undetected in the absence of testing. Even if individuals got tested regularly, the differences in reporting behavior between vaccinated and unvaccinated individuals could have introduced a bias: we saw that monthly questionnaires were less frequently answered by unvaccinated individuals compared to vaccinated individuals, possibly leading to the underreporting of tests, symptoms, and infections in this group (reporting bias). Apart from reporting behavior, it is also possible that risk taking, and thus the risk of becoming infected, was higher among vaccinated individuals than among unvaccinated individuals [40], leading to an increasing number of infections as vaccine protection declined.

In the eCOV study cohort, the calculated VE against symptomatic infection after the second dose of BNT162b2 was generally lower than protection against infection of any severity. This is unexpected and contradicts the current body of evidence from other studies [2,41,42]. This discrepancy in our data could again be due to underreporting among the unvaccinated participants. While 74% (734/992) of the infections in the individuals who had received 2 doses of BNT162b2 were reportedly symptomatic, only 49.9% (318/637) of the infections in unvaccinated individuals were reportedly symptomatic (chi-square test, *P=*.02). As we know from existing studies with larger sample sizes and different study settings [2,42,34], vaccinated individuals tend to experience fewer symptoms.

Supporting existing observational data that show a protective effect of vaccines against symptomatic infection and hospitalization [2,13,33, 43], we saw lower hospitalization rates among vaccinated individuals.

The top expected AEs, both local and systemic, after vaccination reported by our study participants were minor and aligned with those reported in other studies [2,34,43-45]. Our findings thus support extensive reports from public health authorities worldwide on the safety of COVID-19 vaccines [46-48].

In addition to valuable VE insights, our data also suggest a surveillance capability of our study app in tracking infection waves. The vaccine coverage, test positivity rates, and 7-day incidence estimates in the eCOV study cohort showed comparable trajectories to official surveillance data from public health authorities [31,32]. The test positivity rate for PCR testing was higher in the eCOV study cohort than in the German population. One reason for this might be the greater motivation to report positive tests. Notably, the antigen test positivity rate mirrored the pandemic waves, potentially providing a viable solution for pandemic surveillance at a time when PCR testing is increasingly being replaced by self-swab antigen testing. Evaluating our data on digital pandemic surveillance, we demonstrate that digital study apps can be a feasible and scalable solution for pandemic surveillance. In the future, public health authorities could leverage digital platforms for cost-efficient and fast collection of real-world data, complementing traditional surveillance systems.

In summary, our real-world VE data emphasize the importance of continued vaccination efforts in mitigating the impact of pandemics. Overall, the digital collection of self-reported data proved to be a viable tool for digital pandemic surveillance.

### Limitations

It is essential to acknowledge potential biases when interpreting the self-reported data from our self-selected digital cohort. We did not collect data on ethnicity but assumed that the cohort was majority White, similar to the German population, and therefore lacked ethnic diversity. Our cohort was heterogeneous and not representative of the German normal population, with 70.72% (6568/9287) of the participants identifying as women and 38.87% (3610/9287) hailing from the health care sector. Nevertheless, health care workers in our cohort did not report more tests than non–health care workers and did not show increased infection rates. Individuals with a positive COVID-19 test might have had a higher probability to enroll in the study, possibly leading to an overestimation of COVID-19 cases in our cohort [49].

While all our recruitment efforts were aimed at recruiting a representative cohort, we were limited by the nature of a web-based study. Digital literacy bias may have influenced testing behavior because those with higher digital literacy may have been more inclined to use digital platforms for seeking testing and may have been more inclined to report positive tests in the study app. By contrast, individuals with lower digital literacy may have faced barriers in reporting their symptoms or infections through digital means. This bias can skew the data, potentially overestimating the true prevalence of symptoms and infections in the study population. This bias may further have inadvertently excluded marginalized populations and low-income groups, as they are less likely to be digitally literate. Especially for generalizable public health interventions and policies, it is important to assess and acknowledge the needs of these populations.

Moreover, participatory research may attract health-literate users who might behave differently to the general population [50]. Furthermore, health literacy and health-seeking behavior are known to be higher in women than in men [51]. Given that most of the participants were women, our study sample might have been more proactive in seeking information and adhering to preventive measures, potentially leading to an overestimation of VE in our cohort. To improve the generalizability of future research, efforts should be made to include a more diverse and representative sample as well as participants with varying degrees of health literacy, digital literacy, and socioeconomic backgrounds.

Regarding retention, we observed a significant number of dropouts after week 1 of the study (4263/9287, 45.9%). We also observed that answer rates to questionnaires were the highest within 48 hours of email notification. Given that the majority of participants accessed the study app on their mobile devices, transitioning from a web application to a native app could allow for push notifications, potentially enhancing participation rates and retention. Exploring retention further, people who remained in the study beyond the first week stayed on average 129 (SD 114) days, indicating that retention efforts should be intensified in the early weeks after enrollment. To address potential bias, we ran a per-protocol analysis, removing individuals who had dropped out after week 1. The VE analysis still yielded similar results compared to the intention-to-treat approach, where all individuals were included (Figure S15 in Multimedia Appendix 3). Concerning long-term engagement of study participants, survey fatigue may have additionally led to underreporting of symptoms and tests, therefore leading to an underestimation of SARS-CoV-2 infections [52]. Narrowing the definition of primary outcomes and reducing the number of survey questions could help decrease survey fatigue and increase retention rates [53].

Due to the observational nature of our data, we are unable to draw definitive conclusions regarding VE. The low number of reported hospitalizations further limited our ability to calculate VE against hospitalization. Moreover, reports of AEs after vaccination were purely observational and did not allow for causal interpretation.

### Conclusions

We successfully collected self-reported data on the effectiveness of COVID-19 vaccines, COVID-19 testing behavior, symptoms, and AEs after vaccination. The VE in our study cohort was comparable to that in previously published literature. Our data indicate that vaccinations are safe and effective, and third-dose vaccinations partially restore protection against infection. Overall, our data suggest that immunity against SARS-CoV-2 infection in a young, healthy, digital-literate, and predominantly women cohort wanes substantially 24 weeks after primary vaccination series with BNT162b2. Our open study design with self-enrollment led to a nonrepresentative cohort, limiting the generalizability of our study findings. Nevertheless, the data from our cohort showed comparable pandemic trajectories to official surveillance data from public health authorities, highlighting the potential of digital methods to capture the course of the pandemic from real-world data. By applying increased efforts to improve diversity in study cohorts and effectively addressing biases in self-reported data, policy makers could make use of a continuous longitudinal collection of real-world data on pandemic surveillance and VE to inform vaccination strategies. Larger sample sizes and controlled enrollment may be needed to establish causal relationships and estimate VE against specific outcomes such as hospitalization. To improve participation rates, it can be helpful to remind participants about the study through push notifications with short questionnaires that minimize survey fatigue.

Despite its limitations, our study illustrates the potential of easily scalable participatory public health tools. Serving as a novel addition to the public health infrastructure, these tools have the capacity to aid in monitoring real-world VE and enhancing national disease surveillance systems.

## Appendix

Multimedia Appendix 1 The STROBE (Strengthening the Reporting of Observational Studies in Epidemiology) statement (checklist of items that should be included in reports of cohort studies).

Multimedia Appendix 2 Examples of different marketing campaigns used to promote the COVID-19 study on vaccinated and unvaccinated subjects over 16 years (eCOV study).

Multimedia Appendix 3 Supplementary figures.

Multimedia Appendix 4 Overview of study questionnaires.

Multimedia Appendix 5 Vaccine brand combination in primary vaccination series.

Multimedia Appendix 6 Results of vaccine effectiveness against symptomatic infection in the COVID-19 study on vaccinated and unvaccinated subjects over 16 years (eCOV study) cohort.

Multimedia Appendix 7 Symptoms reported during symptomatic SARS-CoV-2 infection, split by vaccination status and SARS-CoV-2 variant.

Multimedia Appendix 8 Distribution of medical treatment and hospitalization during SARS-CoV-2 infection, split by vaccine status and SARS-CoV-2 variant.

Multimedia Appendix 9 Distribution of vaccine brands for respondents who received a third vaccine dose (booster).

Multimedia Appendix 10 Descriptive statistics for entire sample, split by infection status.

## Abbreviations

AE: adverse event
eCOV study: COVID-19 study on vaccinated and unvaccinated subjects over 16 years
PCR: polymerase chain reaction
STROBE: Strengthening the Reporting of Observational Studies in Epidemiology
VE: vaccine effectiveness

## Data Availability

The data sets generated and analyzed during this study are available from the corresponding author on reasonable request.
