# Supplementary material for: COVID-19 Vaccine Effectiveness and Digital Pandemic Surveillance in Germany (eCOV Study): Web Application–Based Prospective Observational Cohort Study
Source: J Med Internet Res. 2024 Jun 4;26:e47070. doi: 10.2196/47070 (PMC11185909; doi:10.2196/47070)
Supplement: Multimedia Appendix 2 [file jmir_v26i1e47070_app2.docx]

**Multimedia Appendix 2**

**Content:** Examples of different marketing campaigns, eCOV study

**Publication title:** COVID-19 Vaccine Effectiveness and Digital Pandemic Surveillance in Germany (eCOV Study): Web-App-Based Prospective Observational Cohort Stud

**Journal:** Journal of Medical Internet Research

**Authors**: Anna-Lena Lang, Nils Hohmuth, Vukašin Višković, Stefan Konigorski, Felix Balzer, Cornelius Remschmidt, Rasmus Leistner.

**Corresponding author:** Anna-Lena Lang, email: annalena.lang.26@gmail.com, phone: +4915756025551

**
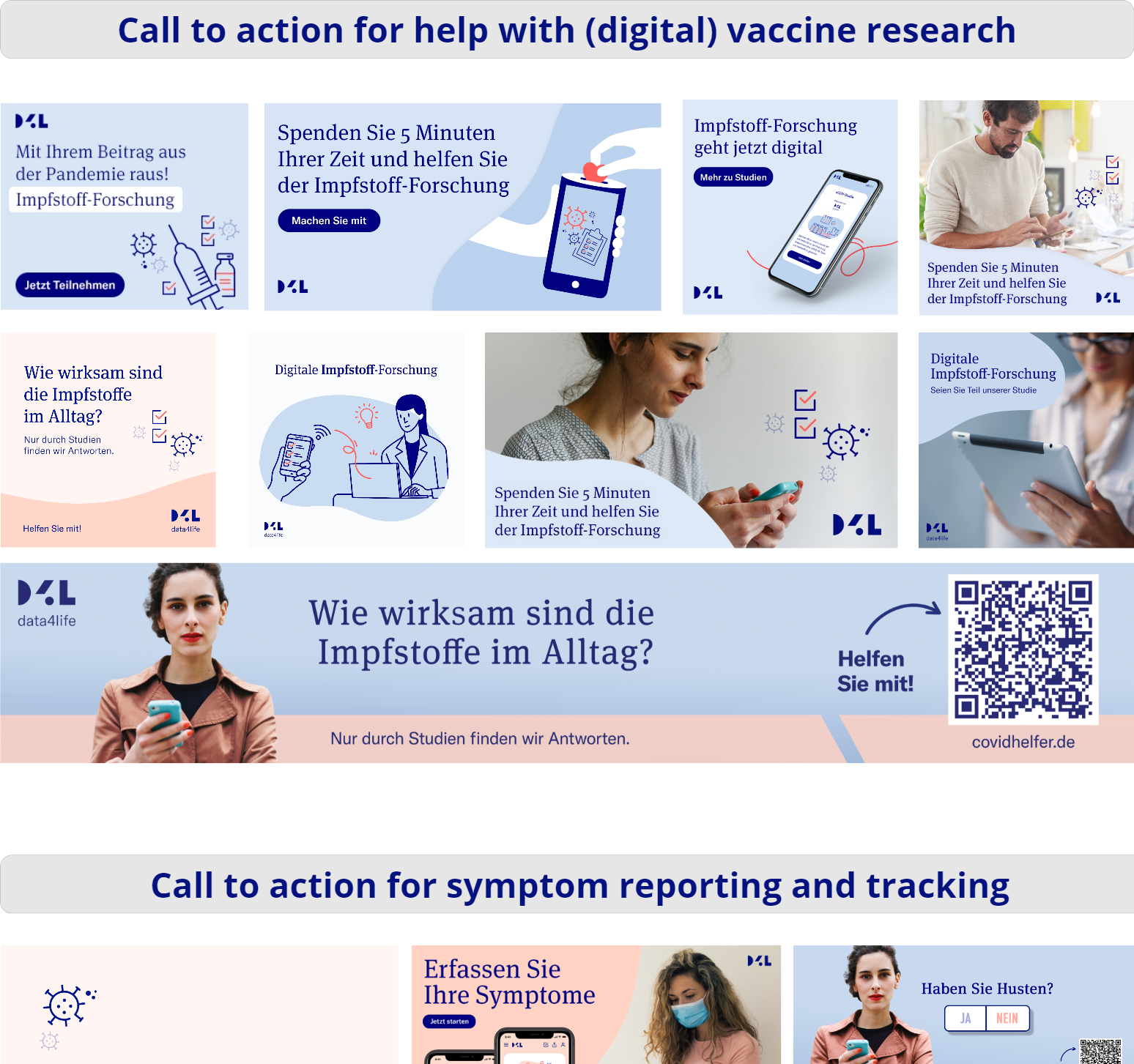

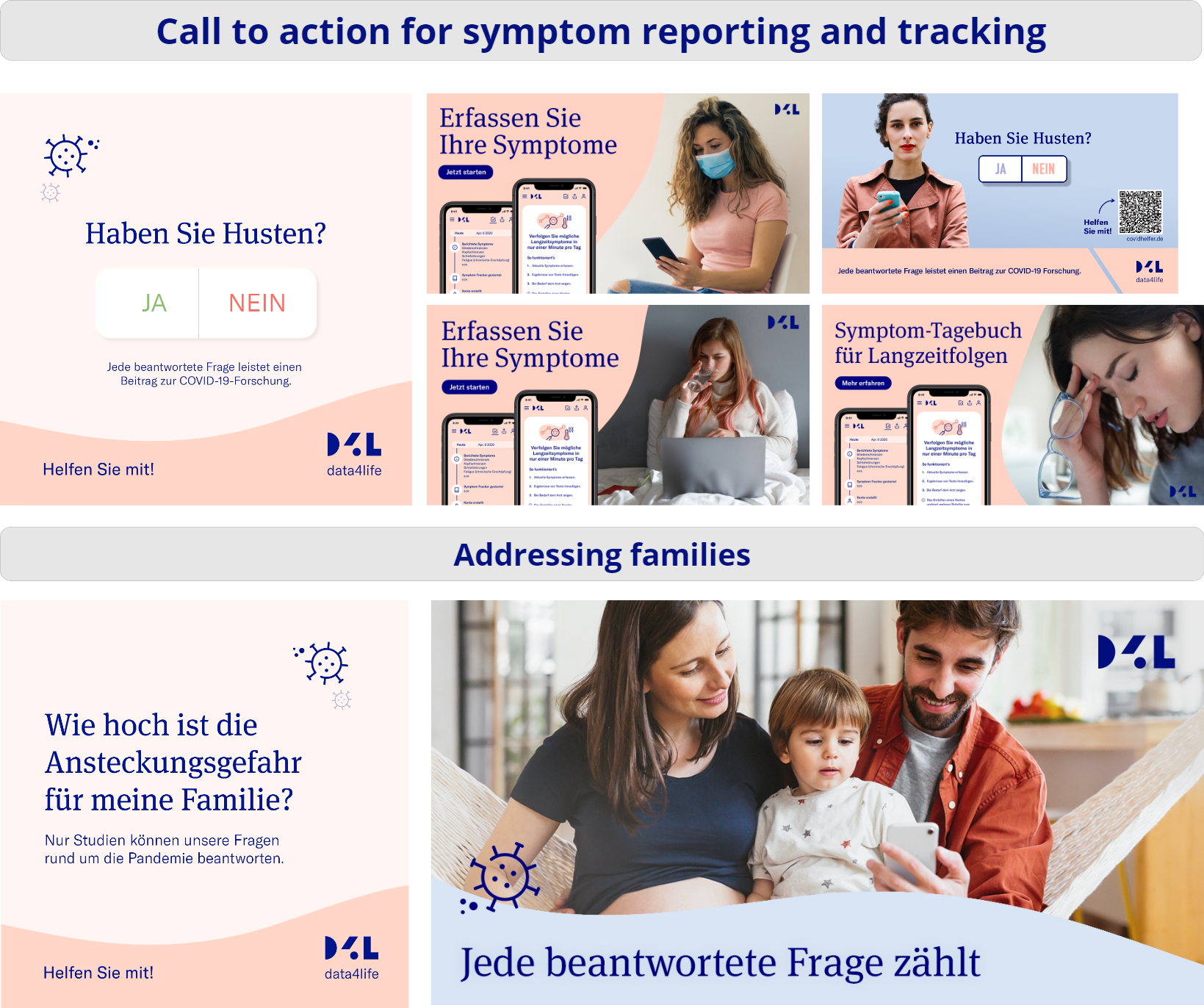

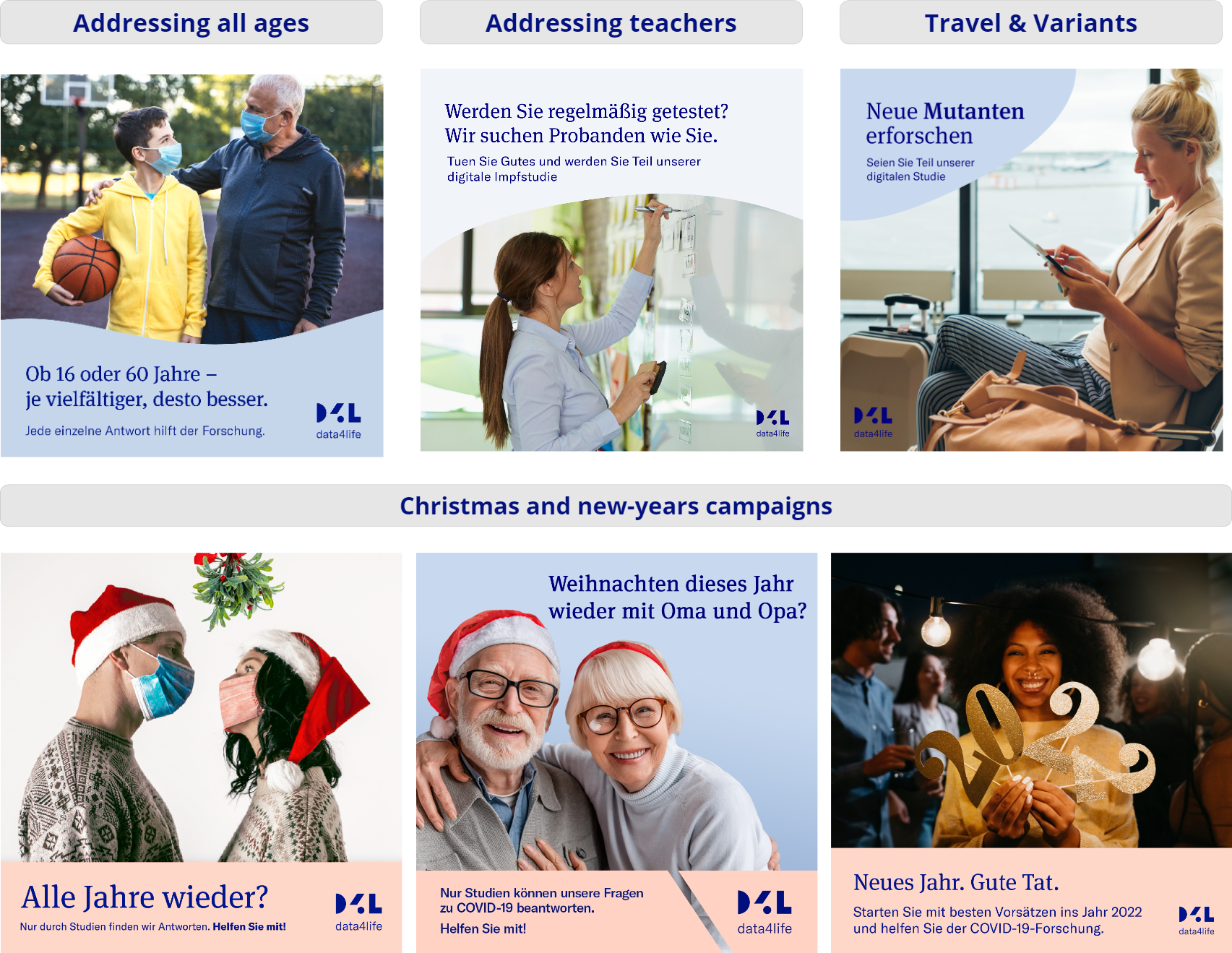

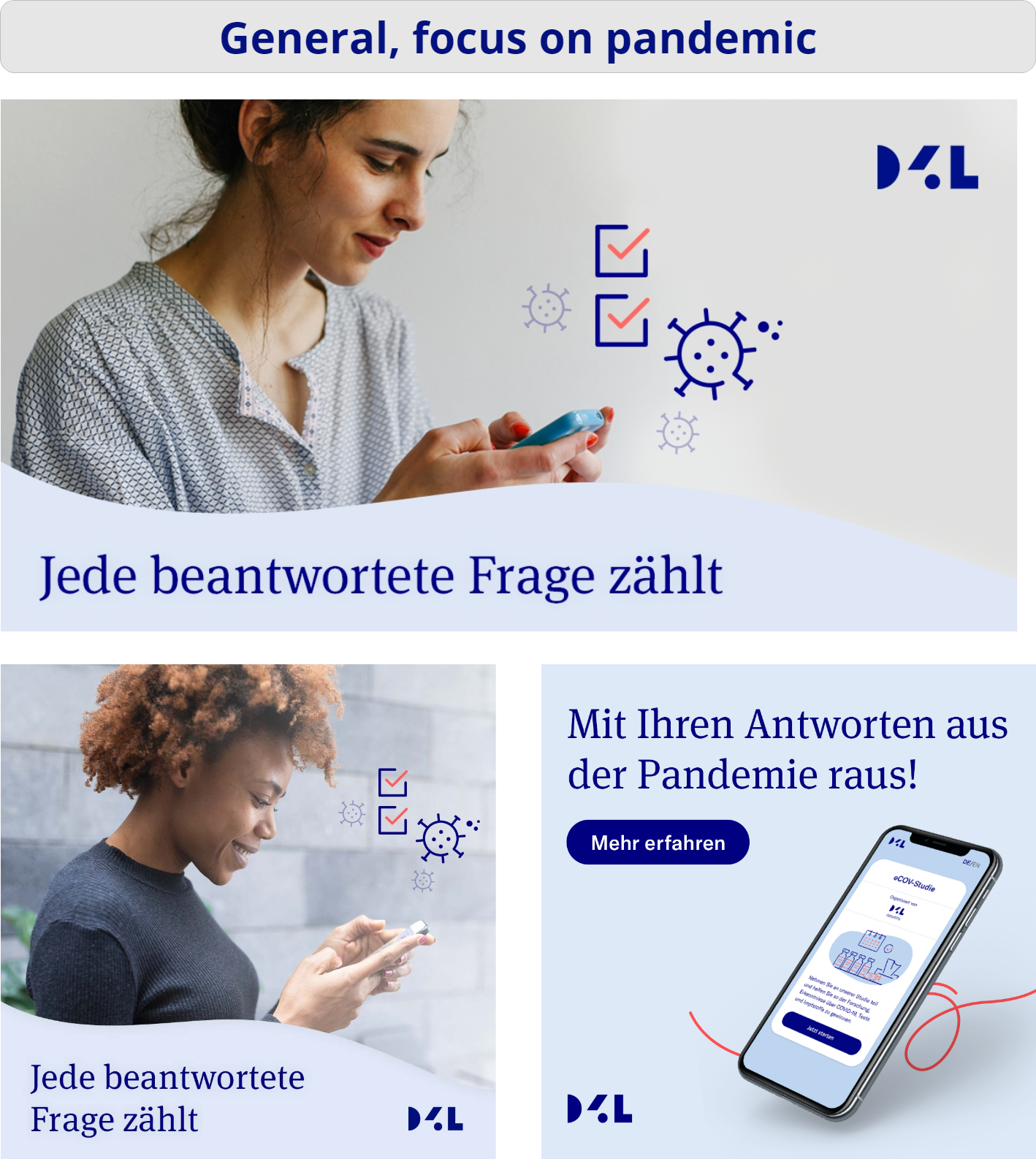
**
