# Supplementary material for: COVID-19 Vaccine Effectiveness and Digital Pandemic Surveillance in Germany (eCOV Study): Web Application–Based Prospective Observational Cohort Study
Source: J Med Internet Res. 2024 Jun 4;26:e47070. doi: 10.2196/47070 (PMC11185909; doi:10.2196/47070)
Supplement: Multimedia Appendix 3 [file jmir_v26i1e47070_app3.docx]

**Multimedia Appendix 3**

**Content:** Supplementary Figures

**Publication title:** COVID-19 Vaccine Effectiveness and Digital Pandemic Surveillance in Germany (eCOV Study): App-Based Prospective Observational Cohort Study

**Journal:** Journal of Medical Internet Research

**Authors**: Anna-Lena Lang, Nils Hohmuth, Vukašin Višković, Stefan Konigorski, Stefan Scholz, Felix Balzer, Cornelius Remschmidt, Rasmus Leistner. Corresponding author: Anna-Lena Lang, email: annalena.lang.26@gmail.com, phone: +4915756025551


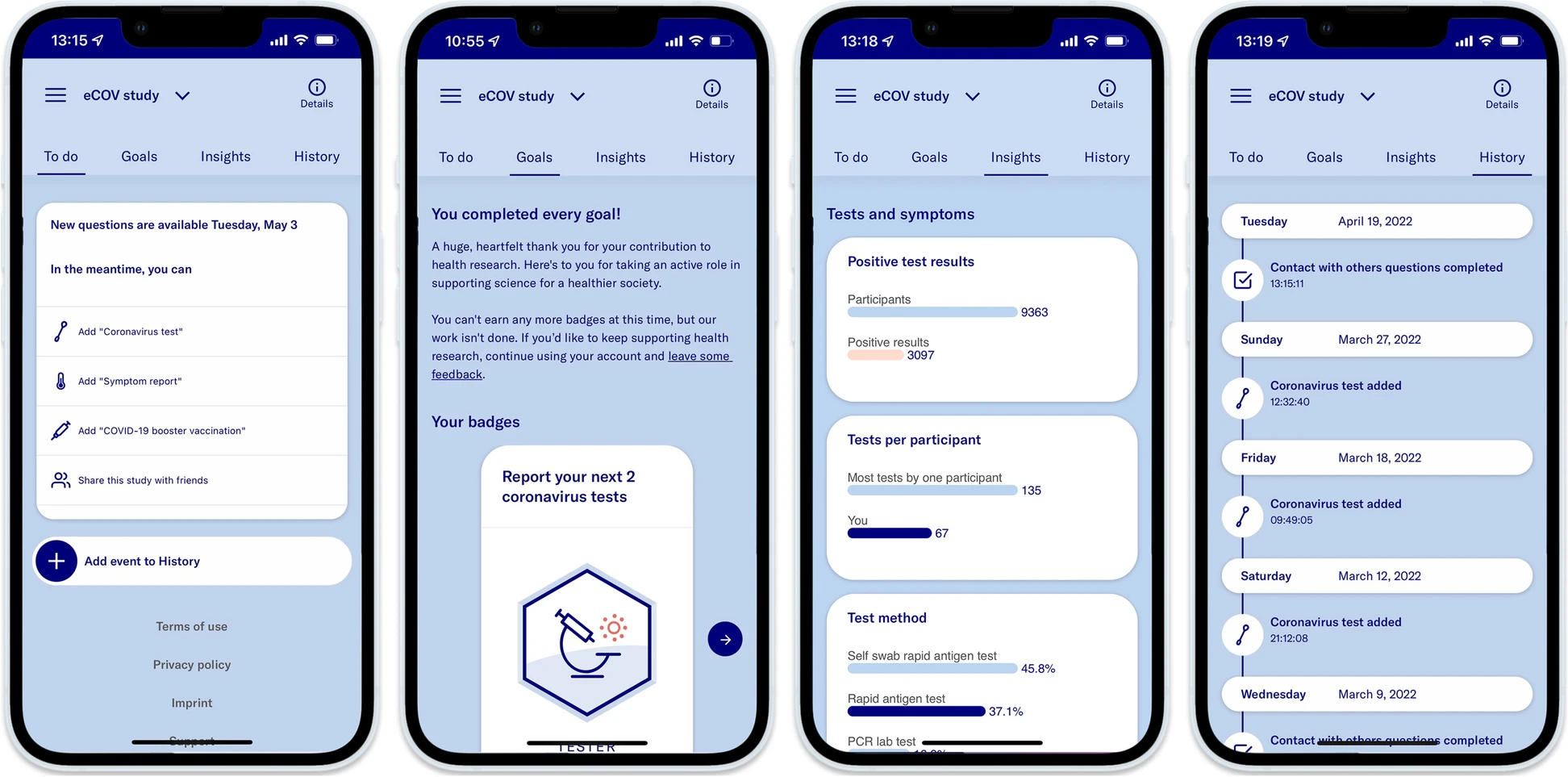


**Figure S1** Screenshots of the study application. This Figure was published as Figure 2 in the first publication of the eCOV study: Hohmuth, N., Khanyaree, I., Lang, AL. et al. Participatory disease surveillance for a mass gathering — a prospective cohort study on COVID-19, Germany 2021. BMC Public Health 22, 2074 (2022). <https://doi.org/10.1186/s12889-022-14505-x>.


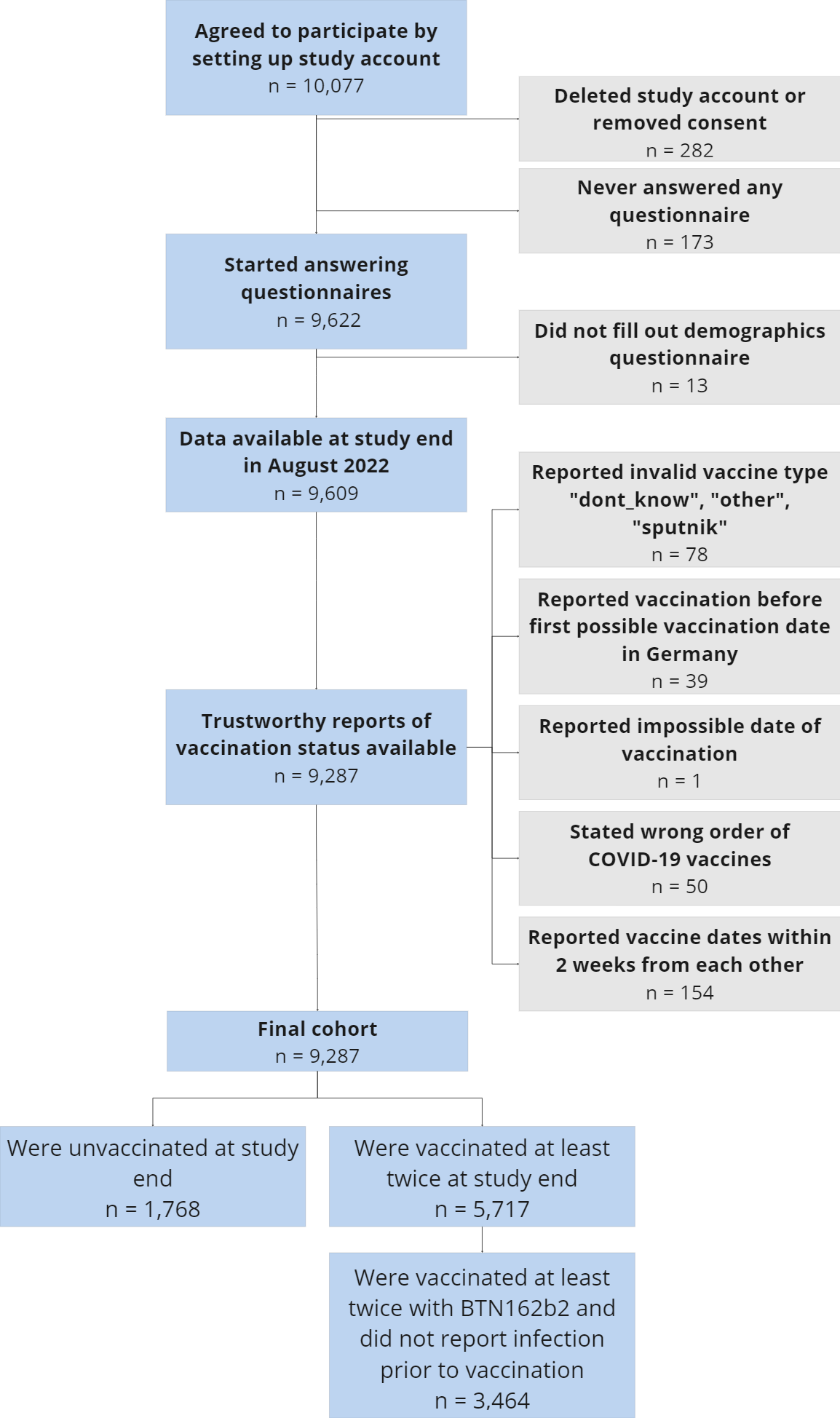


**Figure S2** Flowchart of data filtering process. Quality filtering steps are described in the flow-chart. The final cohort presented in this publication consisted of 9,287 participants.


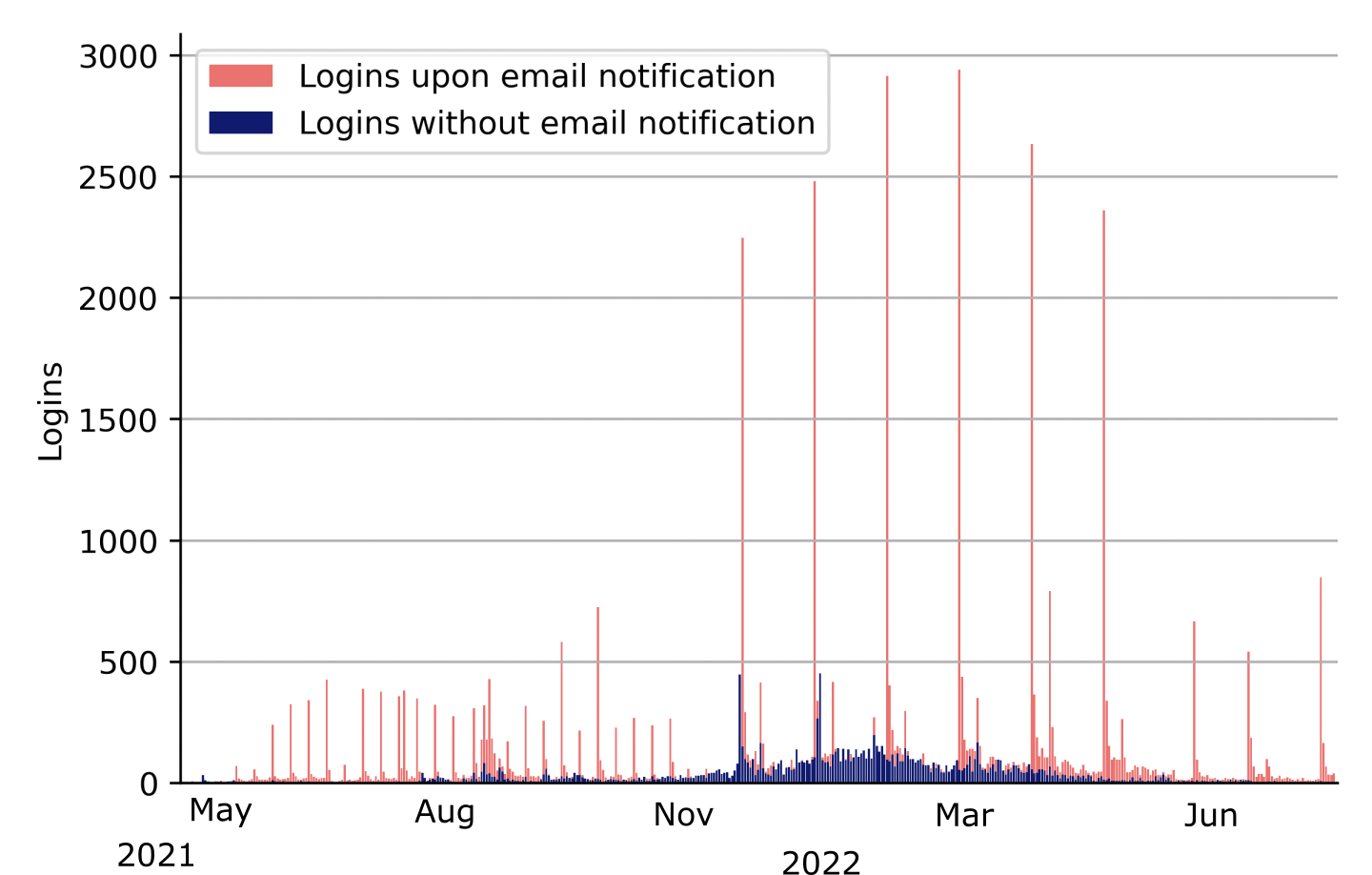


**Figure S3** Logins without and upon email notification over time. Data displayed in this figure was derived from logs generated from the study app. Each bar represents login counts from one day.


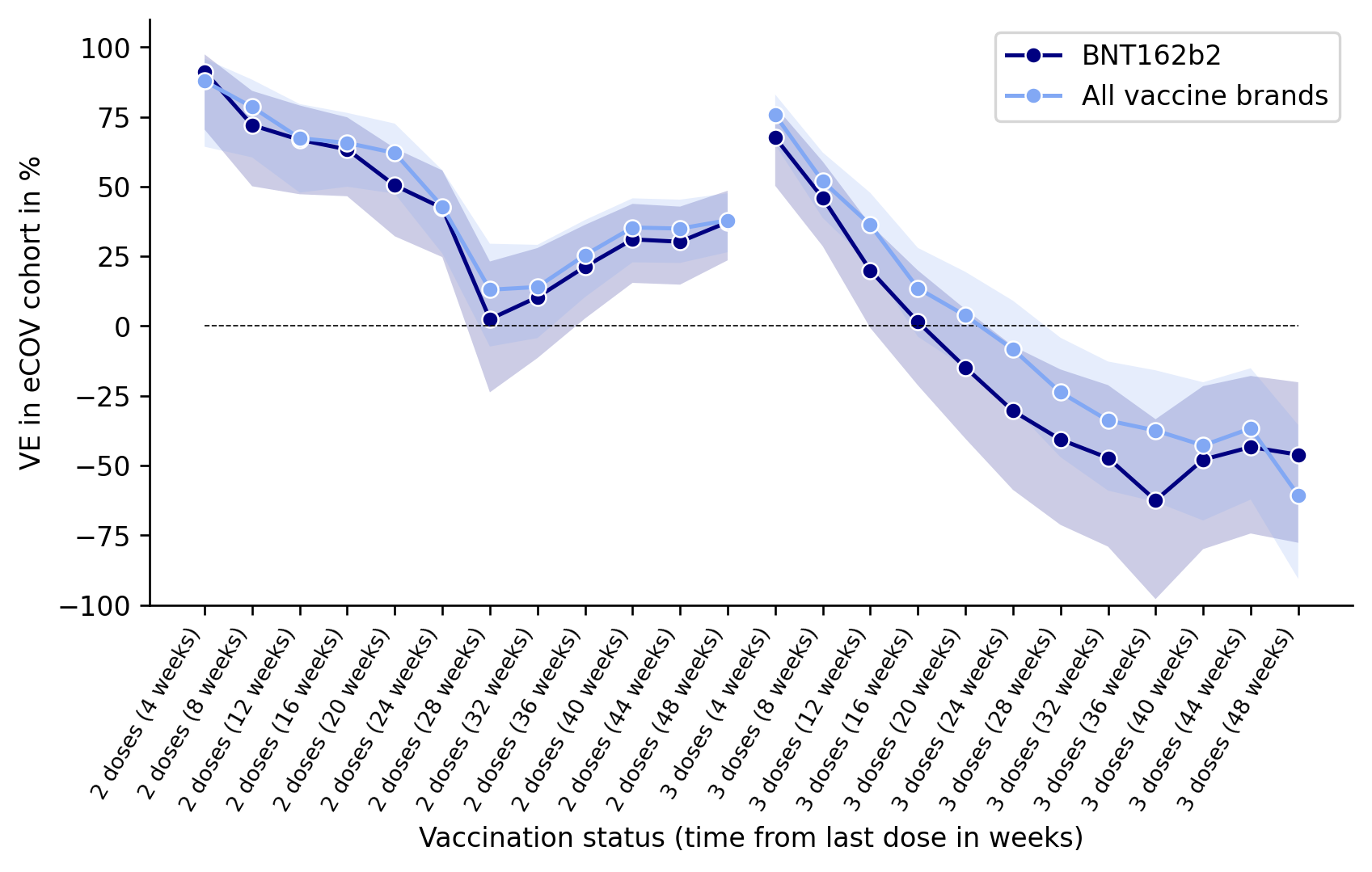


**Figure S4** Vaccine effectiveness against infection of any severity in the eCOV cohort in weeks after the second dose of BNT162b2. VE in % was calculated for weeks 4 to 48 after completing the primary vaccination series with two doses of BNT162b2 and after the third vaccine dose of BNT162b2 for individuals that received all vaccinations with BNT162b2. 95% Confidence intervals are displayed as shaded areas. Abbreviations: *VE* Vaccine effectiveness.


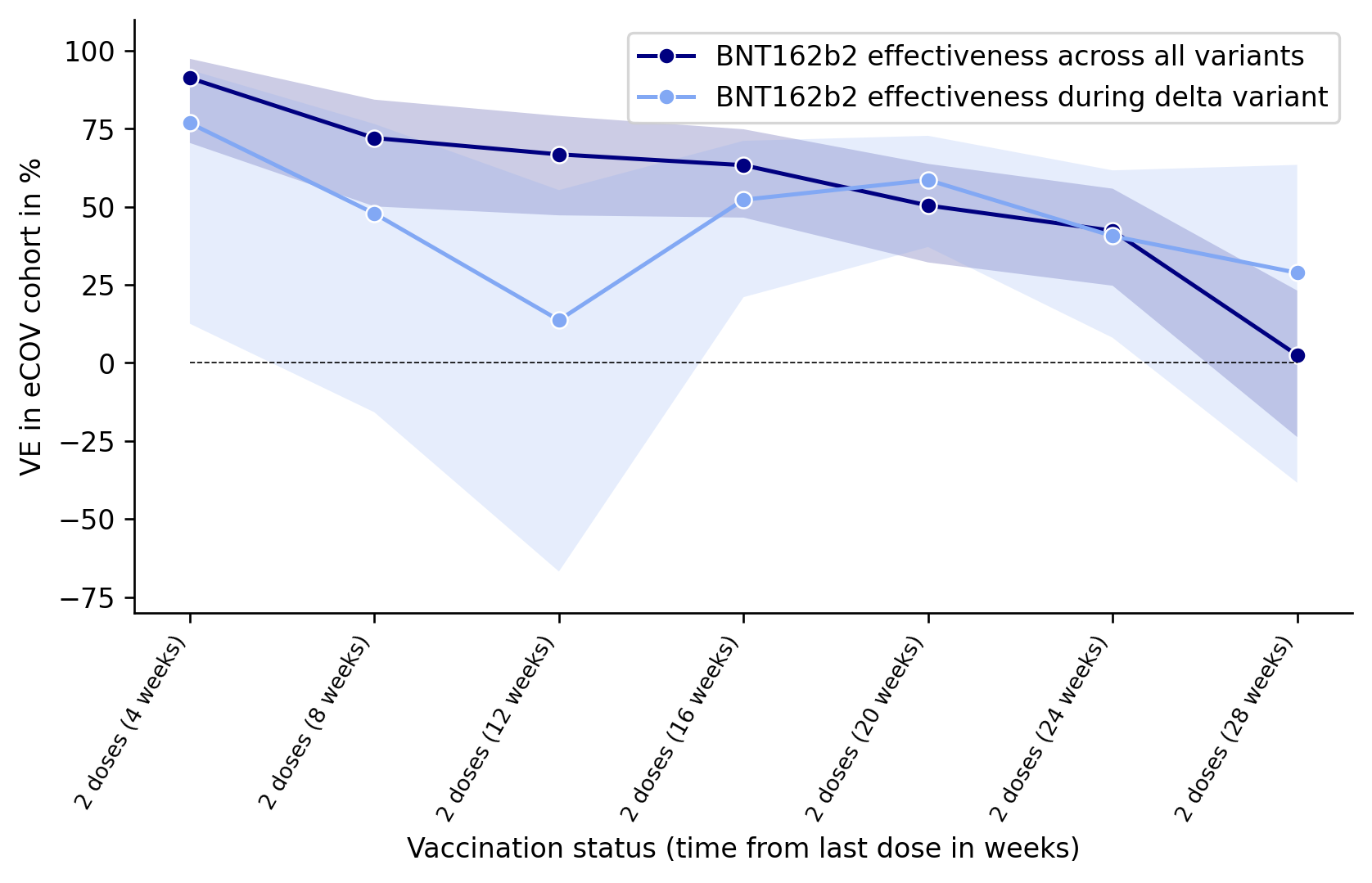


**Figure S5** Vaccine effectiveness during the period of delta variant dominance, against infection of any severity in the eCOV cohort in weeks after the second dose of BNT162b2. VE in % was calculated for weeks 4 to 28 after completing the primary vaccination series with two doses of BNT162b2. 95% Confidence intervals are displayed as shaded areas. Abbreviations: *VE* Vaccine effectiveness.


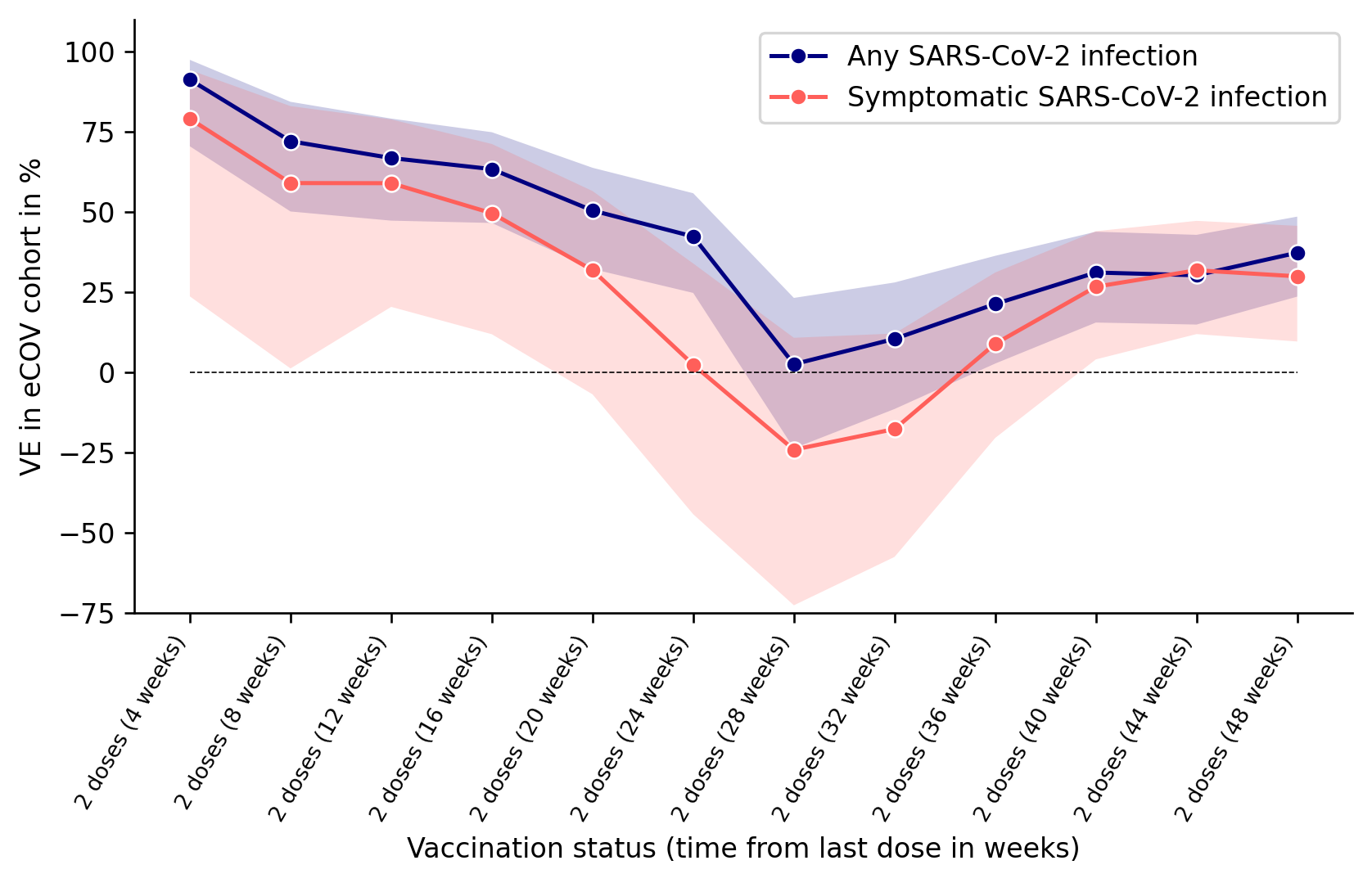


**Figure S6** Vaccine effectiveness against symptomatic infection in the eCOV cohort in weeks after the second dose of BNT162b2. VE in % was calculated for weeks 4 to 48 after completing the primary vaccination series with two doses of BNT162b2. 95% Confidence intervals are displayed as shaded areas. Abbreviations: *VE* Vaccine effectiveness.


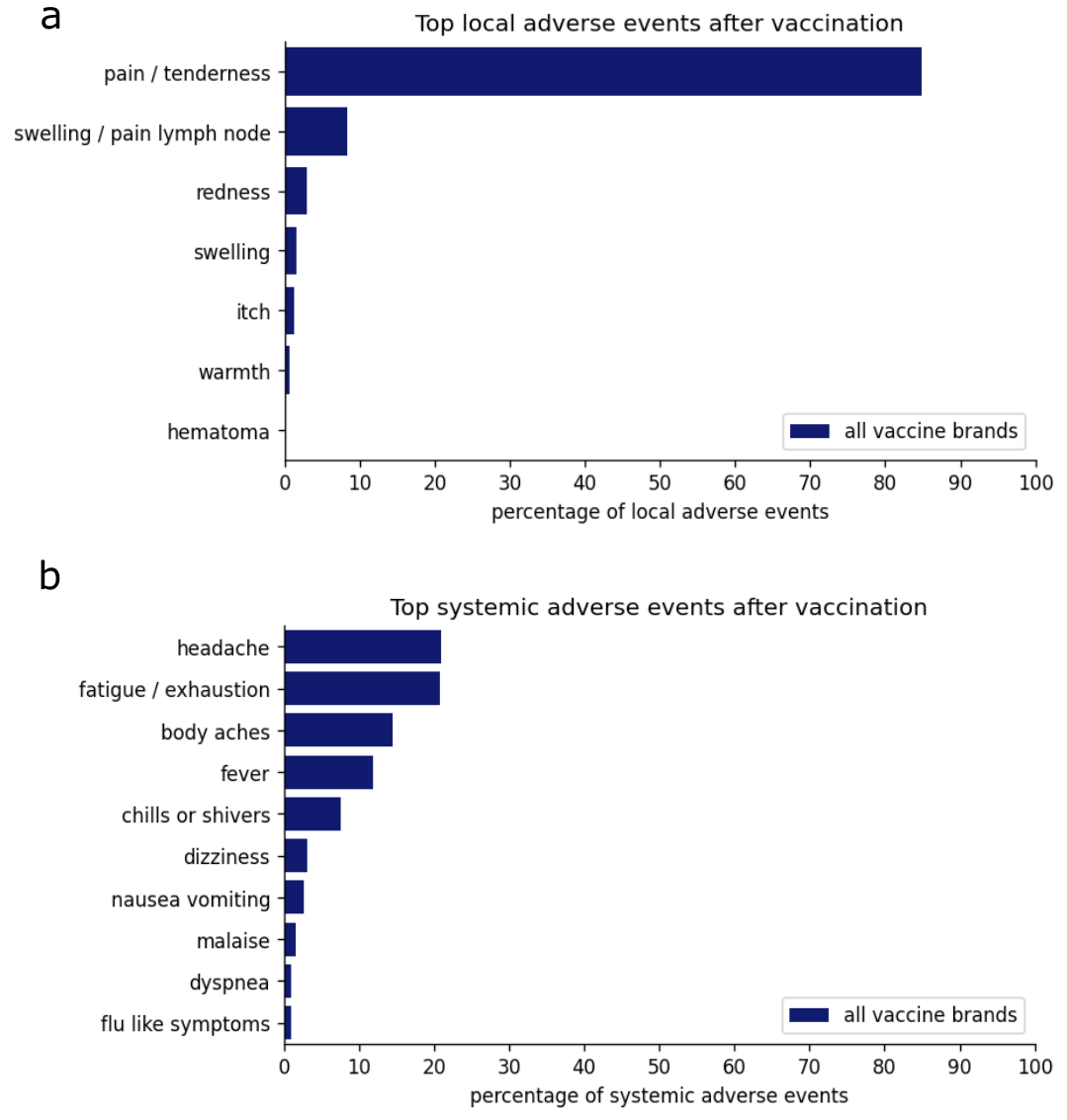


**Figure S7** Barplots visualizing the percentage of adverse events reported in the eCOV cohort after vaccination, all vaccine brands. Percentages reflect proportion of adverse events among all reported adverse events.


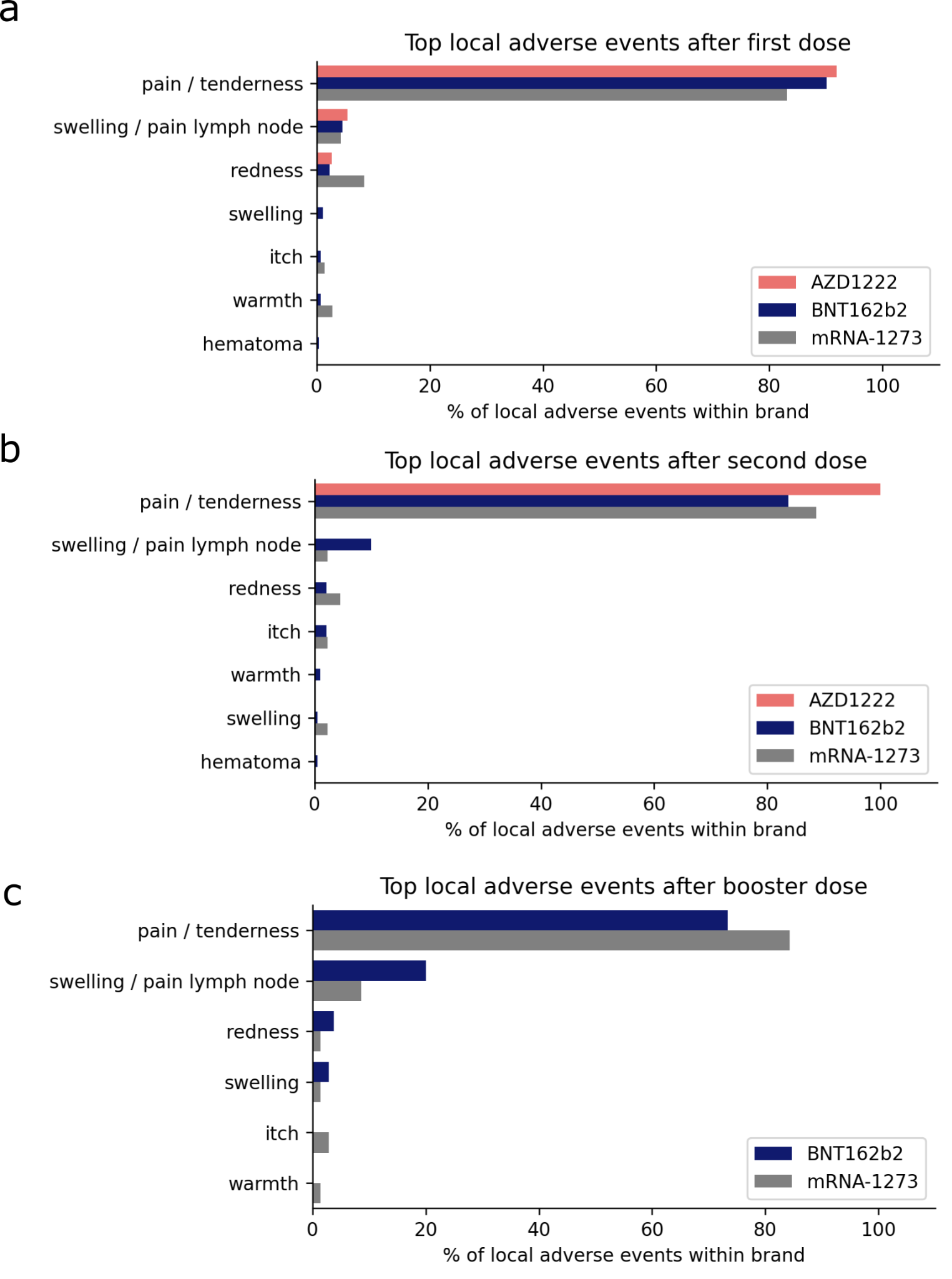


**Figure S8 a-c** Barplots displaying the top local adverse events by vaccine brand. Percentages displayed on the x-axis were calculated within each vaccine brand, reflecting the distribution of adverse events within each brand. Different plots represent adverse events occurring within six weeks of a) first vaccine dose, b) second dose and c) third (booster) vaccine dose.


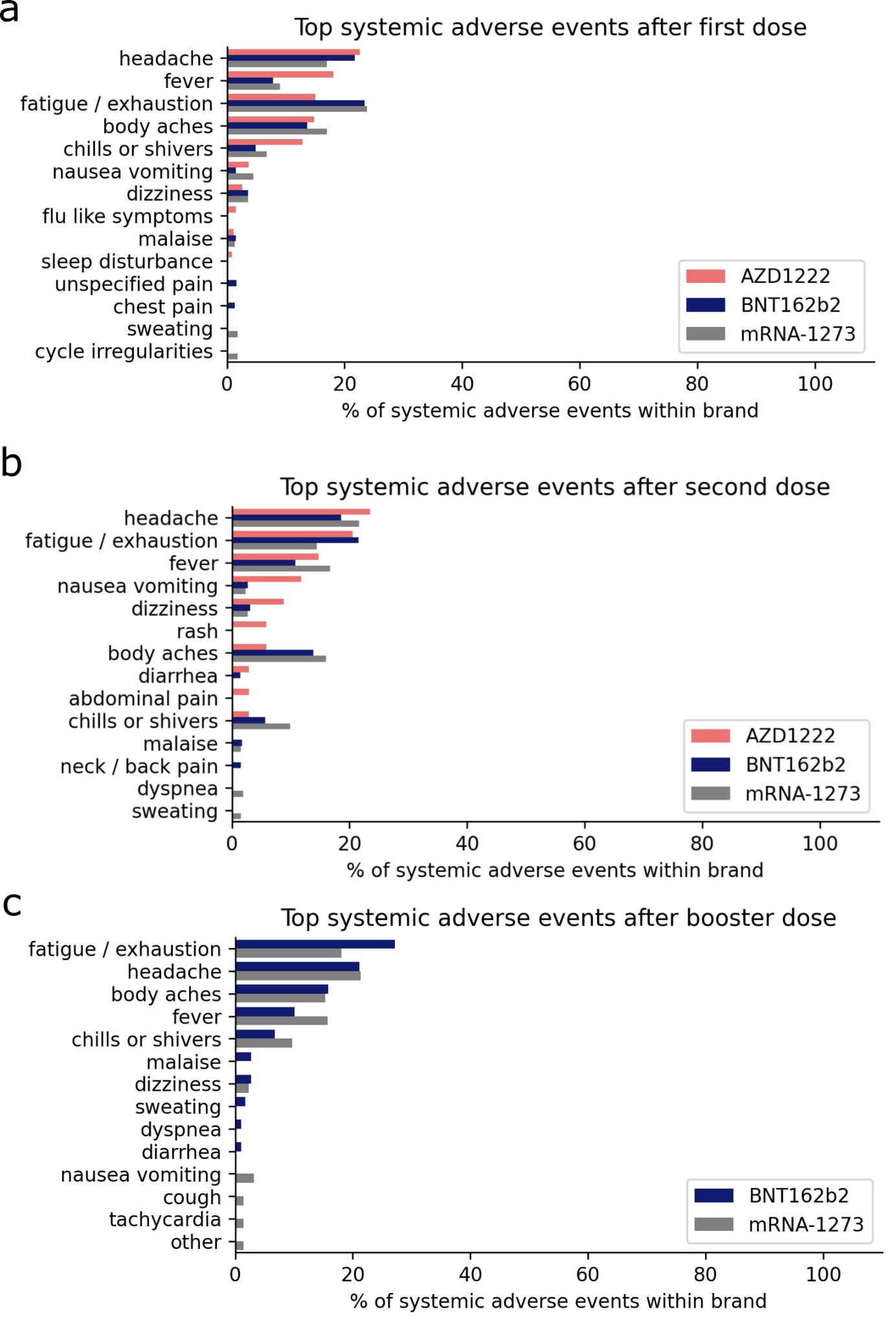


**Figure S9 a-c** Barplots displaying the top systemic adverse events by vaccine brand. Percentages displayed on the x-axis were calculated within each vaccine brand, reflecting the distribution of systemic adverse events within each brand. Different plots represent adverse events occurring within six weeks of a) first vaccine dose, b) second dose and c) third (booster) vaccine dose.


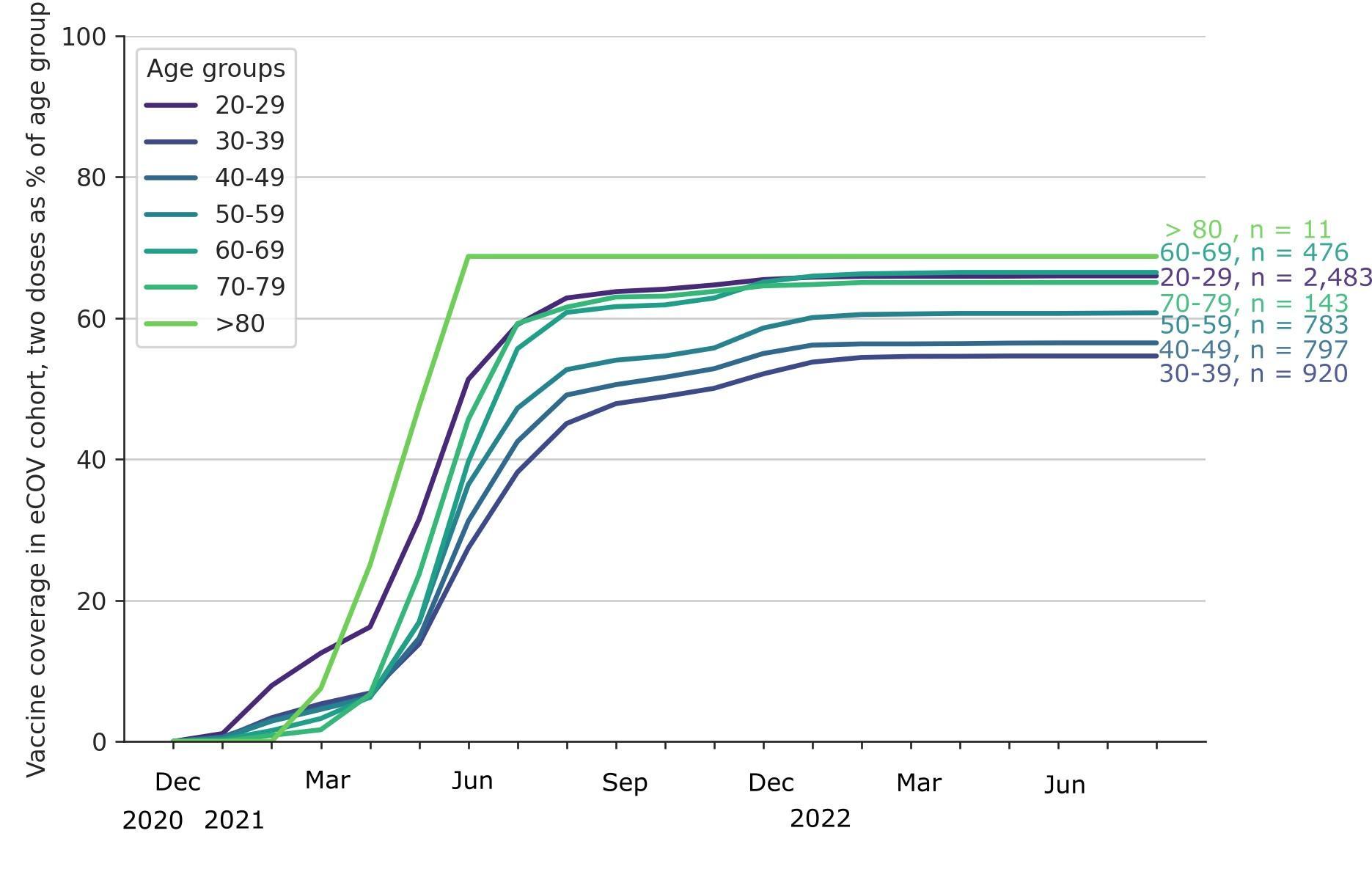


**Figure S10** Line plot visualizing the vaccine coverage for second vaccine dose (any vaccine brand combinations) over time, split by age groups.


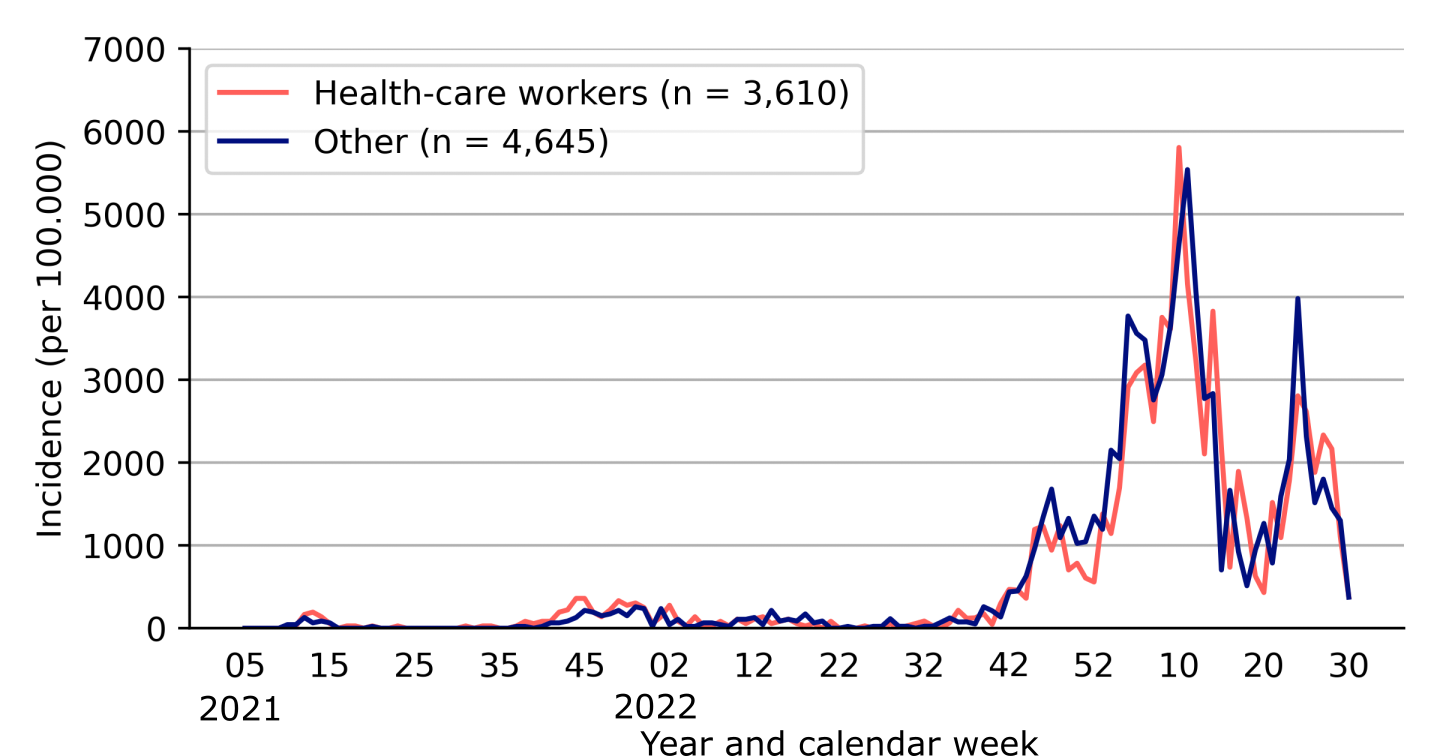


**Figure S11** Comparison of the seven-day incidence per 100.000 people, split by health-care workers and non health-care workers within the eCOV cohort. PCR positive SARS-CoV-2 infections only.


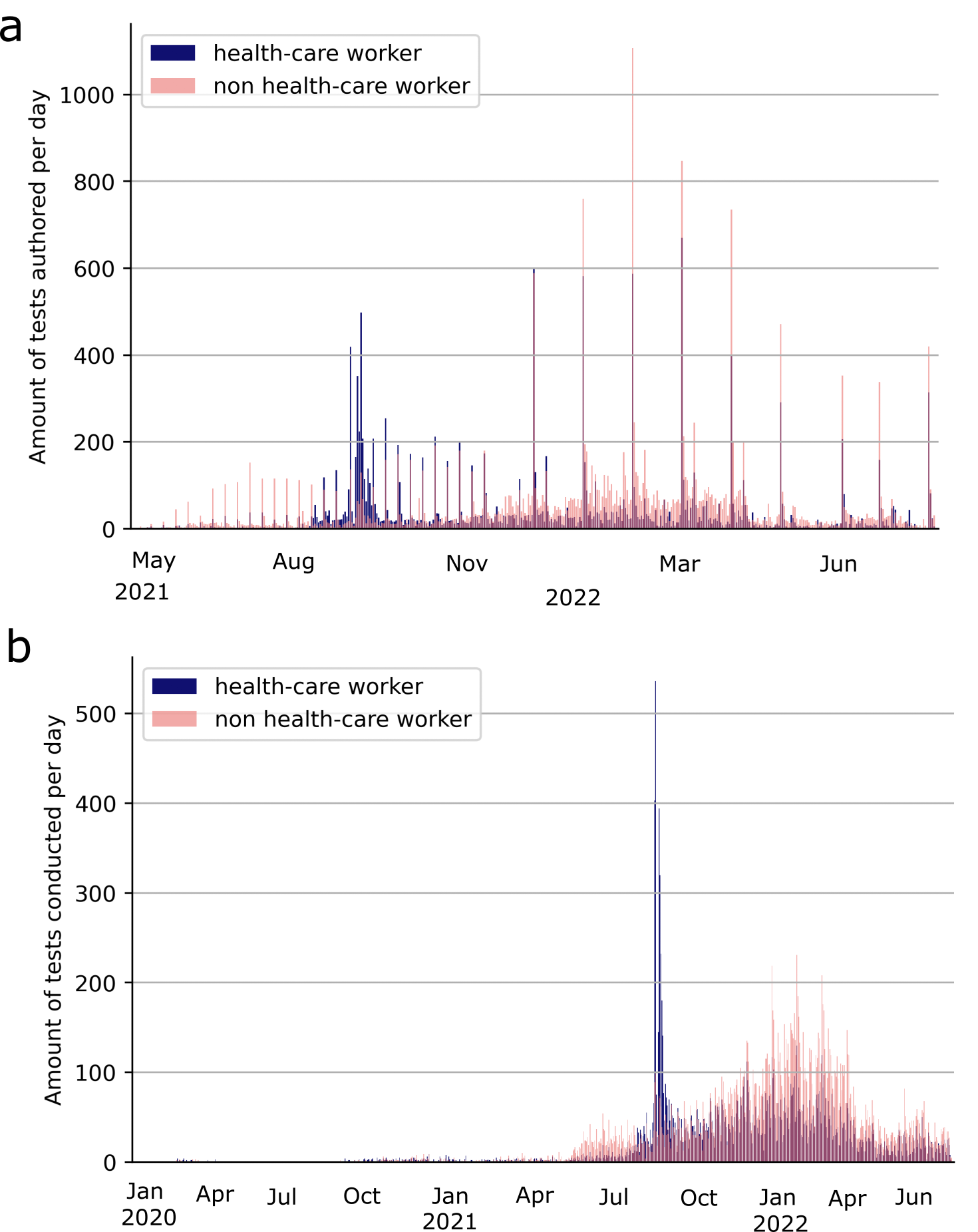


**Figure S12** Overview of reported tests split by health-care worker status. a) Barplot displaying the amount of tests that were reported in the app per day, as a measure of activity in the study app. b) Barplot displaying the amount of tests that were conducted each day (= day of testing), as a measure of testing behavior of the eCOV cohort. As retrospective data reports were allowed, reported tests date back to as early as January 2020 although the study only started in May 2021.


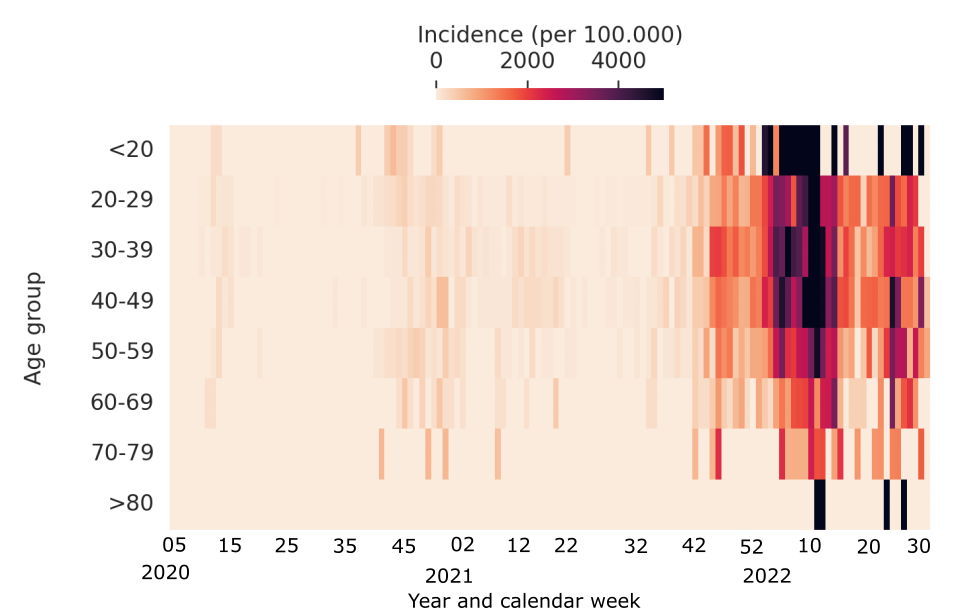


**Figure S13** Heatmap displaying the seven-day incidence of SARS-CoV-2 infections of any severity in the eCOV cohort, stratified by age-group.


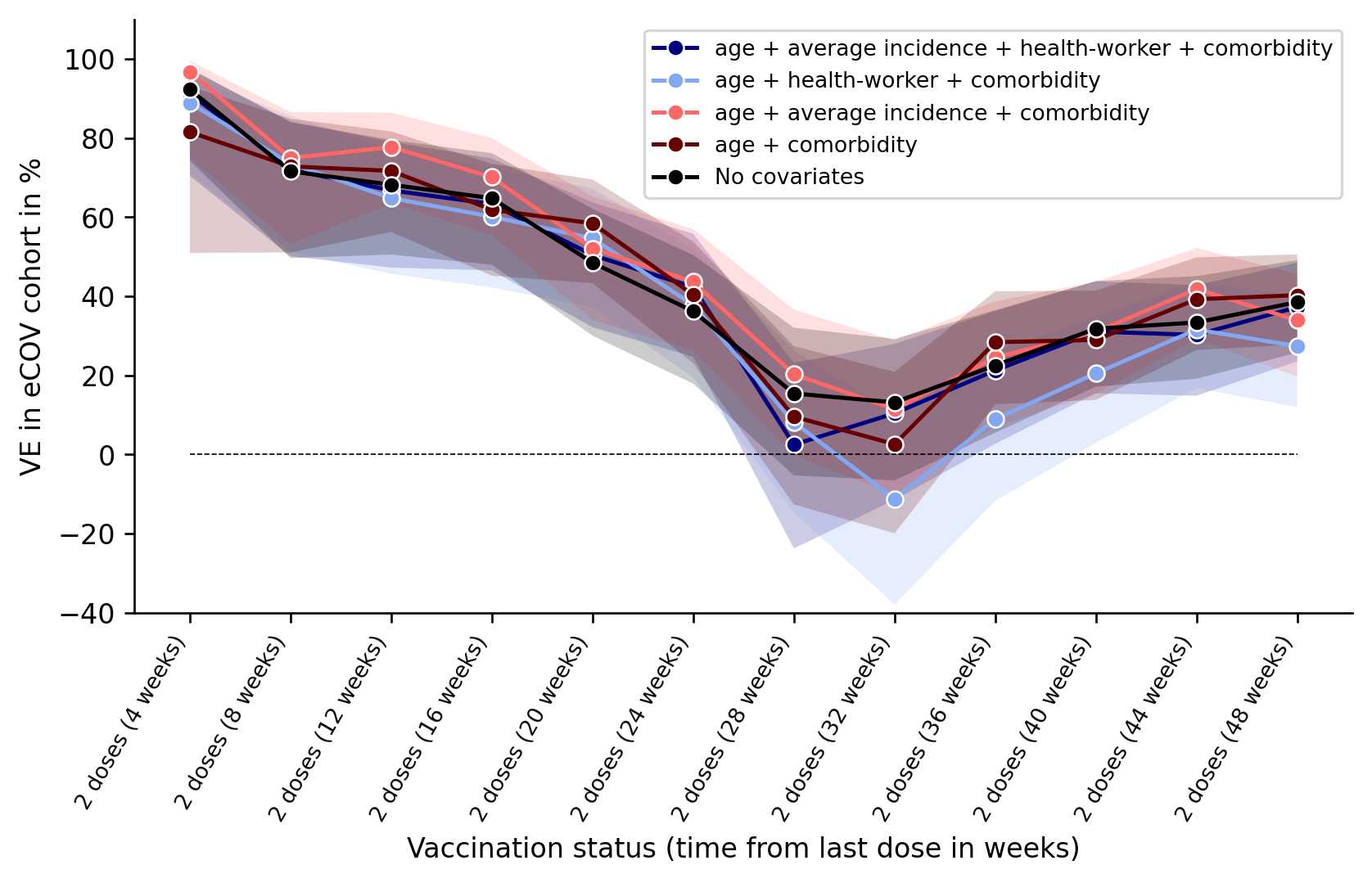


**Figure S14** Vaccine effectiveness against infection of any severity in the eCOV cohort in weeks after the second dose of BNT162b2. Color coding shows results from different logistic regression models with different covariates added to the model. VE in % was calculated for weeks 4 to 48 after completing the primary vaccination series with two doses of BNT162b2. 95% Confidence intervals are displayed as shaded areas. Abbreviations: *VE* Vaccine effectiveness.


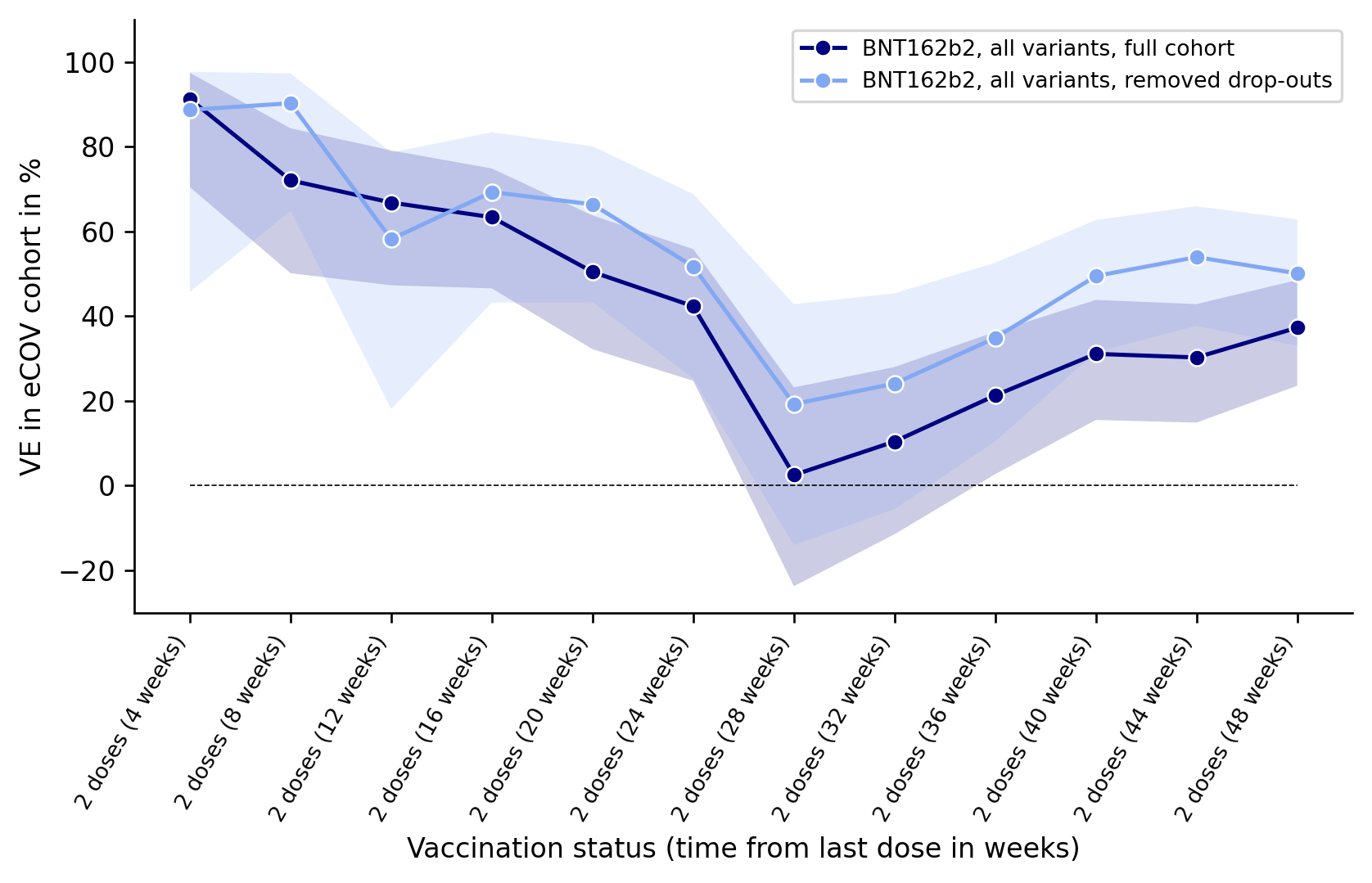


**Figure S15** Vaccine effectiveness against infection of any severity in the eCOV cohort in weeks after the second dose of BNT162b2, comparing VE analysis with and without including drop-outs. Individuals that did not stay in the study for more than 7 days were declared as drop-outs. VE in % was calculated for weeks 4 to 48 after completing the primary vaccination series with two doses of BNT162b2. 95% Confidence intervals are displayed as shaded areas.
